# Supplementary material for: Notch signaling is a driver of glandular stem cell activity and regenerative migration after damage
Source: EMBO J. 2025 Nov 5;45(2):374–93. doi: 10.1038/s44318-025-00607-w (PMC12811363; doi:10.1038/s44318-025-00607-w)
Supplement: Supplementary file 2 — Appendix [file 44318_2025_607_MOESM2_ESM.pdf]

Appendix for "Notch signaling is a driver of glandular stem cell activity and regenerative migration after damage"

Table of contents

Appendix Figure S1 2

Appendix Figure S2 4

Appendix Figure S3 5

Appendix Figure S4 6

Appendix Figure S5 8

Appendix Figure S6 9

Appendix Figure S7 11

Appendix Figure S8 13

Appendix Figure S9 15

Appendix Table S1 16

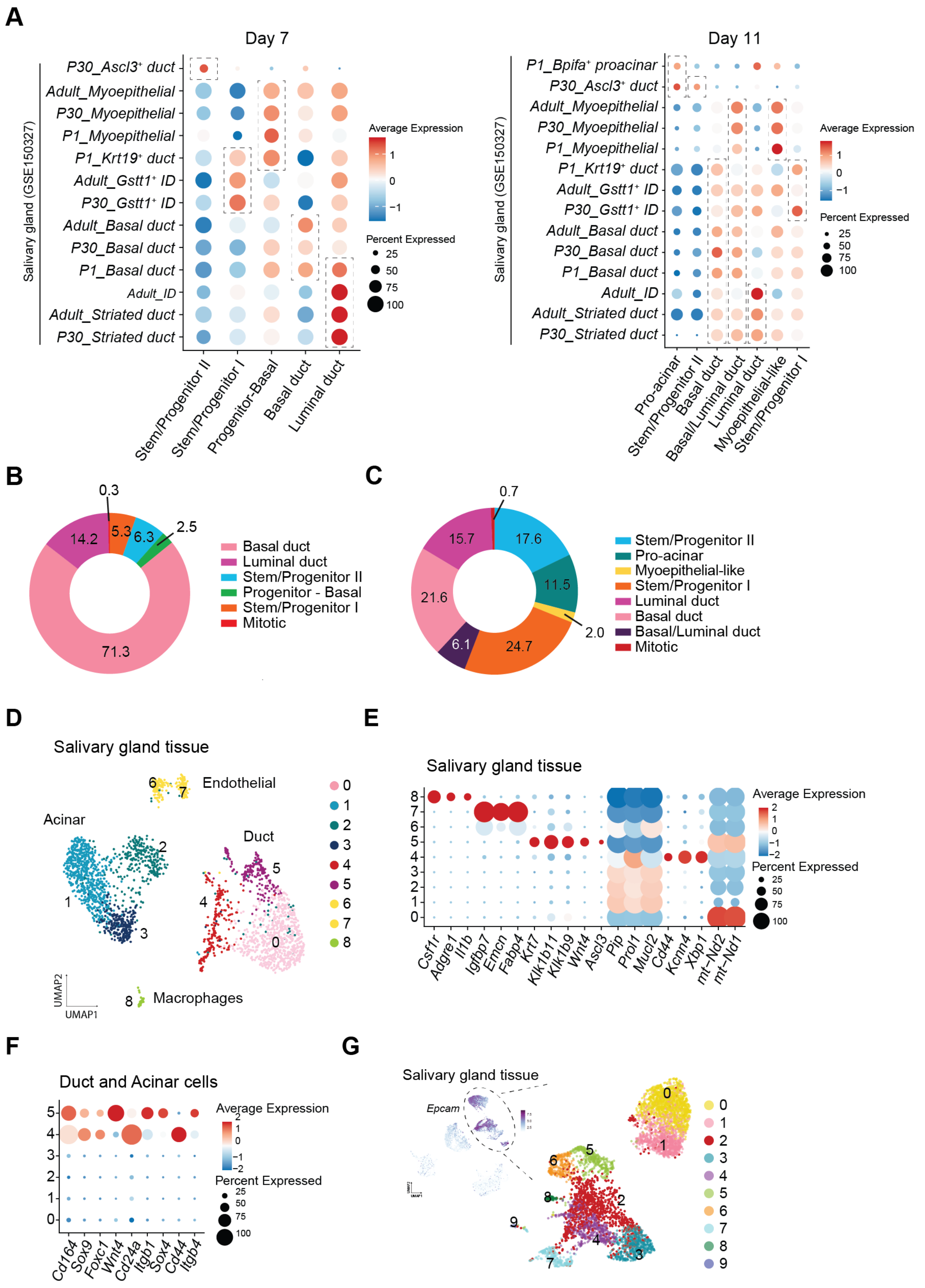

Appendix Figure S1. scRNAseq analysis of mSGOs and salivary gland tissue

(**A**) Dot plot showing the average expression of the top 50 marker genes for each mouse salivary gland population from GSE150327 (Hauser et al., 2020) in 7-day mSGOs (left) and 11-day mSGOs (right). (**B**) Cell proportion of clusters in 7-day mSGOs, UMAP is shown in Figure 1D. (**C**) Cell proportion of clusters in 11-day mSGOs, UMAP is shown in Figure 1E. (**D**) UMAP of the salivary gland tissue dataset showing the main cell

populations. (**E**) Dot plot showing cell type marker genes of the salivary gland tissue dataset. (**F**) Dot plot showing stem/progenitor cell markers extrapolated from 7-day mSGOs in ductal and acinar subpopulations. (**G**) UMAP of the salivary gland tissue dataset from E-MTAB-13374 (McKendrick et al., 2023) showing *Epcam* expression, re-clustering, and the main populations of *Epcam*-expressing cells.

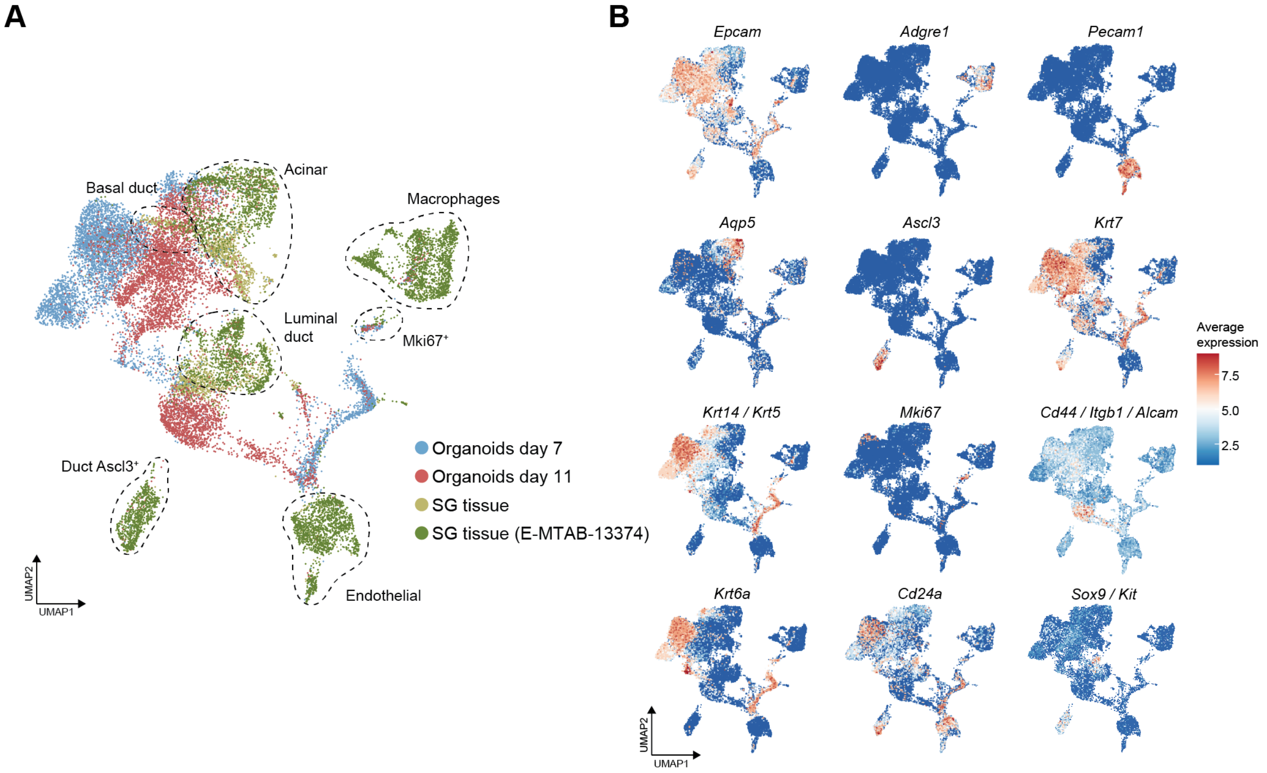

Appendix Figure S2. Integration of mSGO and salivary gland tissue datasets

(**A**) UMAP of the integrated 7-day mSGO, 11-day mSGO, and salivary gland tissue datasets, including the dataset from E-MTAB-13374 (McKendrick et al., 2023), showing the different sources and the main tissue populations. (**B**) Feature plots showing the average expression of *Epcam* (epithelial cells), *Adgre1* (macrophages), *Pecam1* (endothelial cells), *Aqp5* (acinar cells), *Ascl3* (*Ascl3*⁺ duct cells), *Krt7* (duct cells), *Krt14/Krt15* (basal duct cells), *Mki67* (cycling cells), *Cd44/Itgb1/Alcam* (stem/progenitor II cells), *Krt6a* (basal duct cells and stem/progenitor I cells), *Cd24a* (stem/progenitor I cells), *Sox9/Kit* (intercalated duct cells).

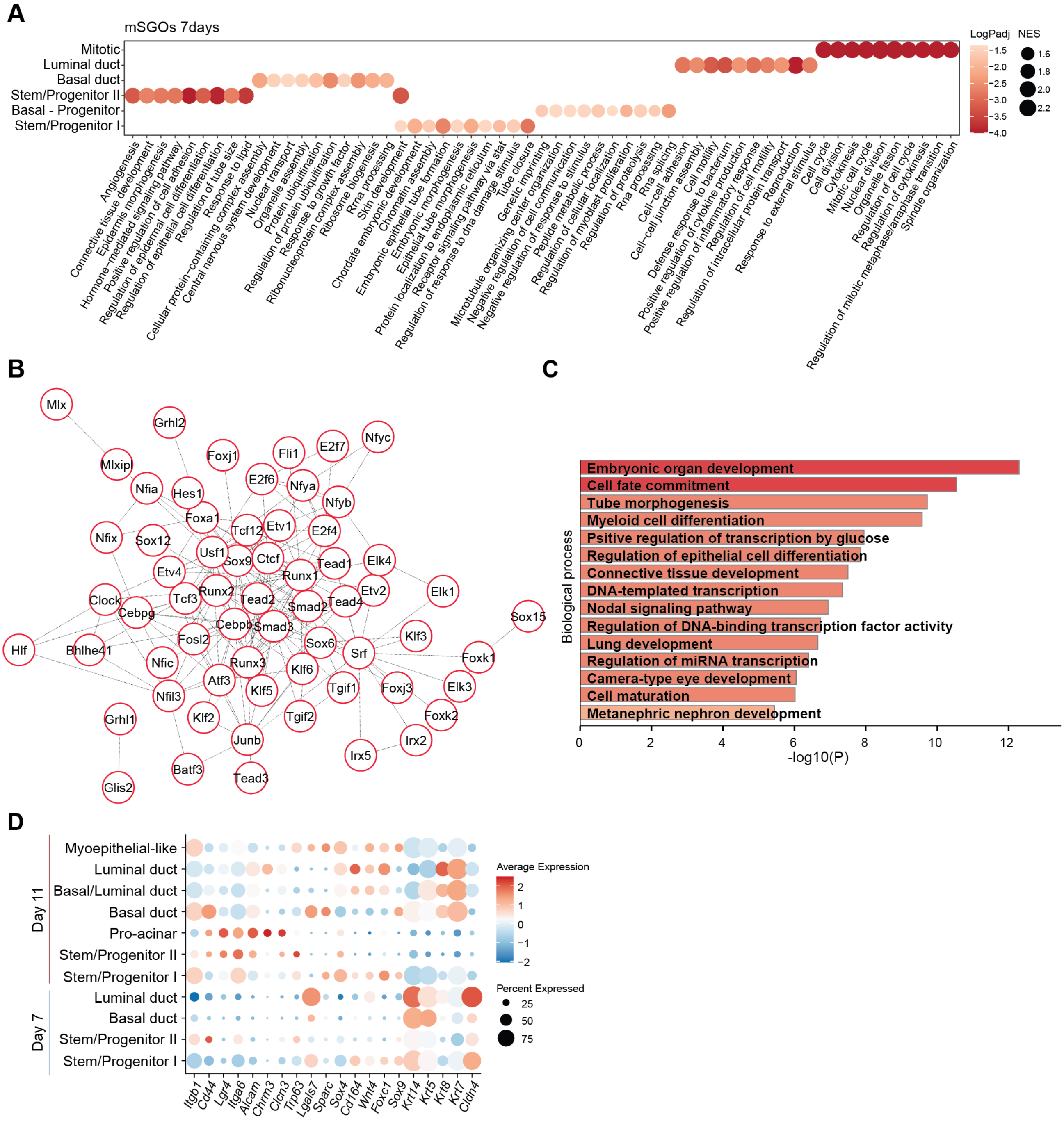

Appendix Figure S3. ATAC-seq and characterization of the 7-day and 11-day mSGO-integrated dataset

(**A**) Dot plot showing the top 10 upregulated biological processes in each population of 7-day mSGOs. (**B**) Network analysis showing transcription factors related to significant ATAC-seq motifs. (**C**) Top 15 biological processes related to ATAC-seq motif transcription factors. (**D**) Dot plot showing cell type marker genes of the 7-day and 11-day mSGO-integrated dataset. UMAP is shown in Fig. 1C.

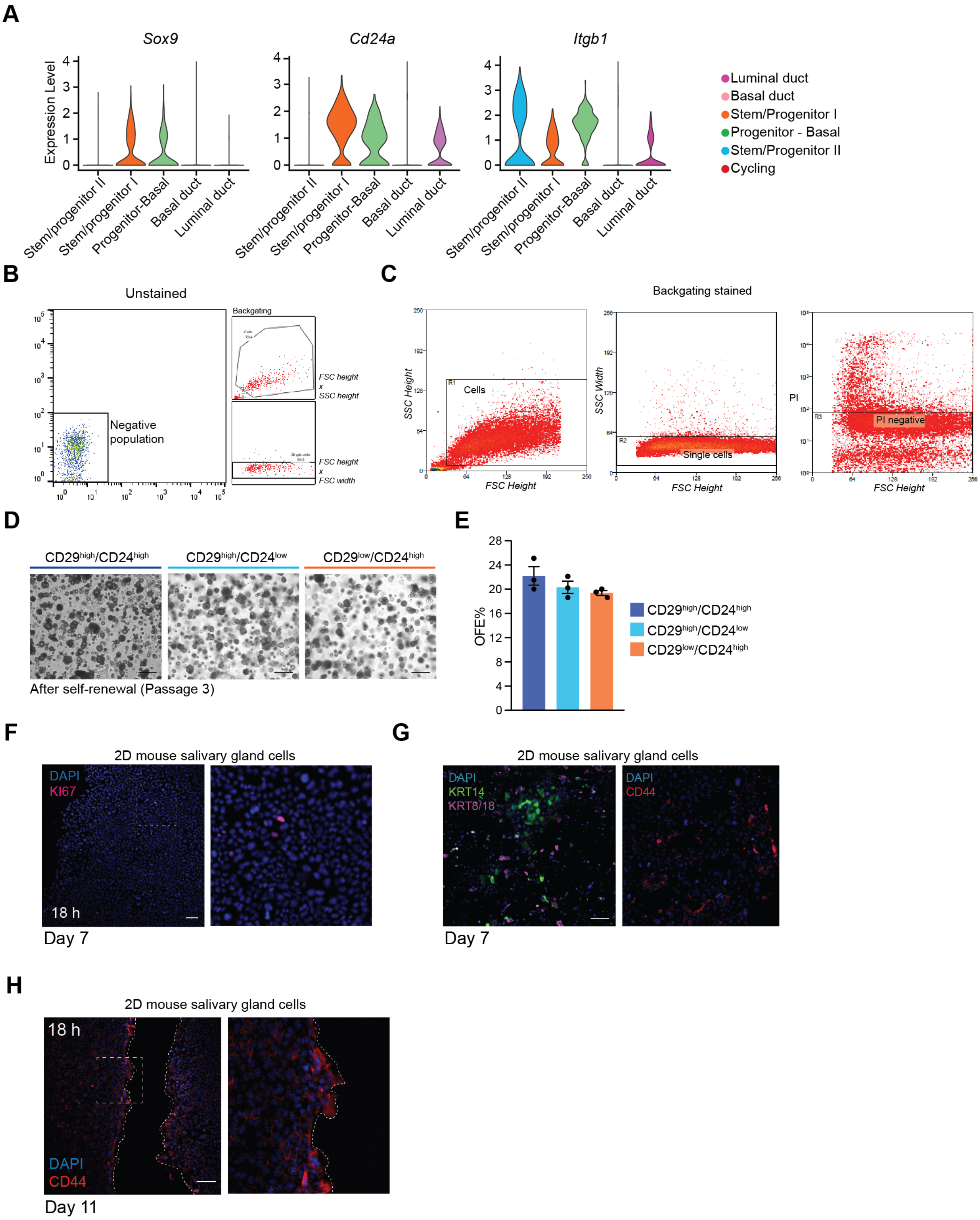

Appendix Figure S4. FACS of 7-day mSGOs and 2D salivary gland cells

(**A**) Violin plot showing expression of *Sox9, Cd24a* (CD24) and *Itgb1* (CD29) across the 7-day mSGO populations. (**B**) Representative FACS plot of an unstained sample using the same gating strategy shown in Figure 2g. (**C**) Backgating of FACS plot shown in Fig. 2G. (**D**) Representative images of sorted cells after self-renewal (P3). Scale bar, 100 µm. (**E**) Organoid quantification of sorted cells after self-renewal (P3) shown as organoid formation efficiency (OFE) (means ± s.e.m; n = 3 animals/condition). (**F**) Representative

immunofluorescence image of 2D salivary gland cells under serum-starvation conditions, 18 hours after wound generation, showing KI67 expression. Scale bar: 100 µm. (**G**) Representative images of immunofluorescence staining of 2D salivary gland cells showing the expression of KRT14, KRT8/18 and CD44. Scale bar, 100 µm. (**H**) Representative immunofluorescence images of salivary gland cells derived from 11-day mSGOs, 18 hours after wound generation, showing CD44 expression. Scale bar, 100 µm.

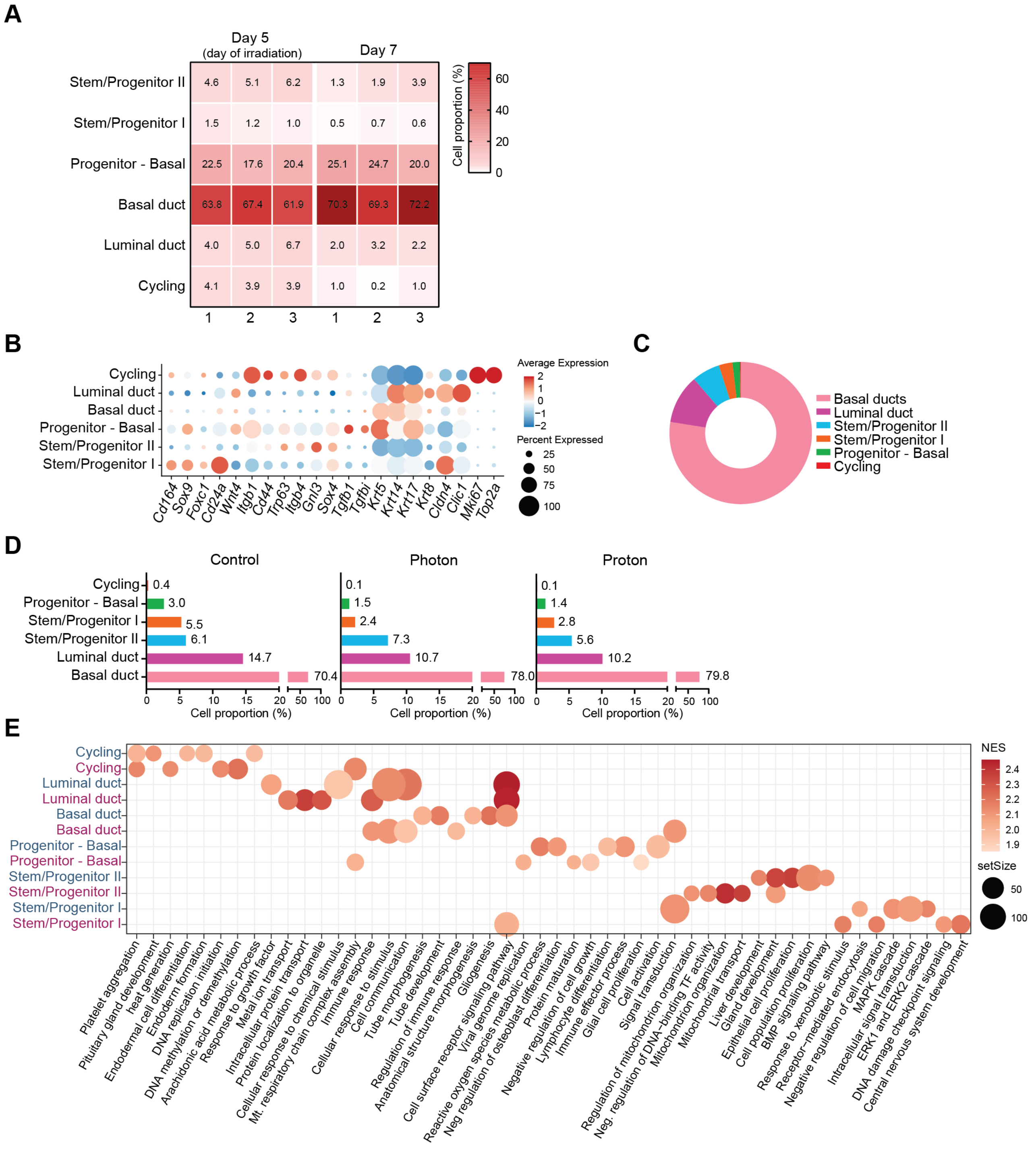

Appendix Figure S5. scRNAseq analysis of photon and proton-irradiated mSGOs
(**A**) Heatmap showing the cell proportions of 5-day and 7-day mSGOs, generated from bulk RNA-seq data and deconvoluted using CIBERSORTx with the 7-day mSGO dataset as a reference. (**B**) Dot plot showing cell marker genes of the merged control, photon and proton dataset. UMAP is shown in Fig. 3C. (**C**) Cell proportion of clusters in the merged control, photon and proton dataset. UMAP is shown in Fig. 3C. (**D**) Bar plot showing the proportion of cells in control, photon and proton datasets. UMAP is shown in Fig. 3C. (**E**) Dot plot showing the top 5 upregulated biological processes in each population of 7-day mSGOs after photon (magenta) and proton (blue) irradiation.

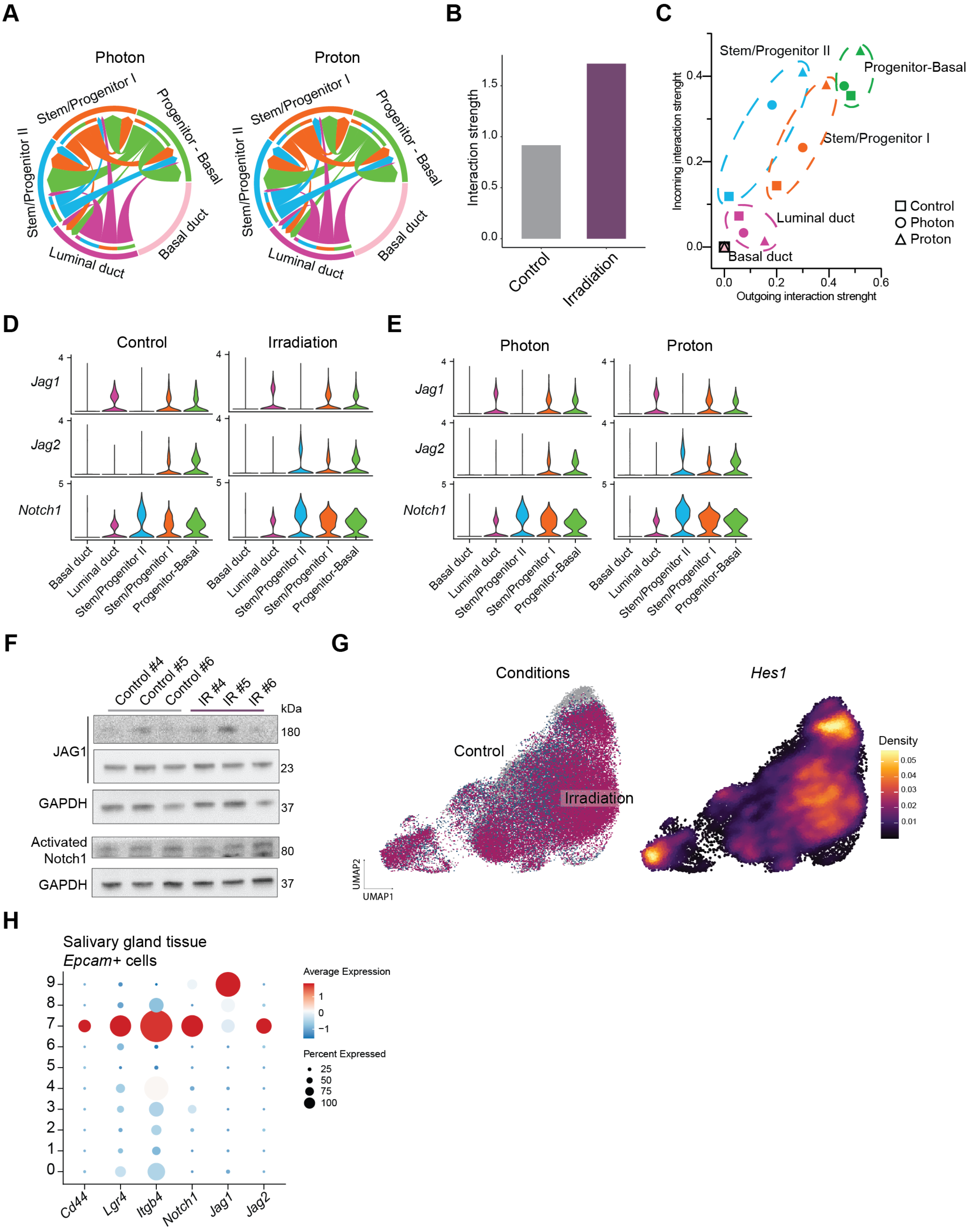

Appendix Figure S6. CellChat analysis and validation of Notch signaling activation

(**A**) Chord plot showing interaction strength in photon and proton-irradiated mSGOs. (**B**) Bar plot showing the total interaction strength in control and irradiation datasets. (**C**) PCA plot showing outgoing and incoming interaction strengths. (**D**) Violin plot showing the expression of Notch-related genes *Notch1, Jag1* and *Jag2* in photon and proton-irradiated mSGO datasets. (**E**) Violin plot showing the expression of Notch-related genes

Notch1, Jag1 and *Jag2* in photon and proton-irradiated mSGO datasets. (**F**) Western blot analysis of JAG1, activated NOTCH1 and GAPDH in control and irradiated mSGOs at day 7. Additional biological replicates are shown in Fig. 3J. (**G**) UMAP showing control and irradiation conditions (left), and density plot showing the average expression of *Hes1* (right). (**H**) Dot plot showing the expression of *Cd44, Lgr4, Itgb4, Notch1, Jag1* and *Jag2* in *Epcam*+ cells extrapolated from E-MTAB-13374 (McKendrick *et al*, 2023). Cluster numbers refer to the UMAP shown in Appendix Fig. S1G.

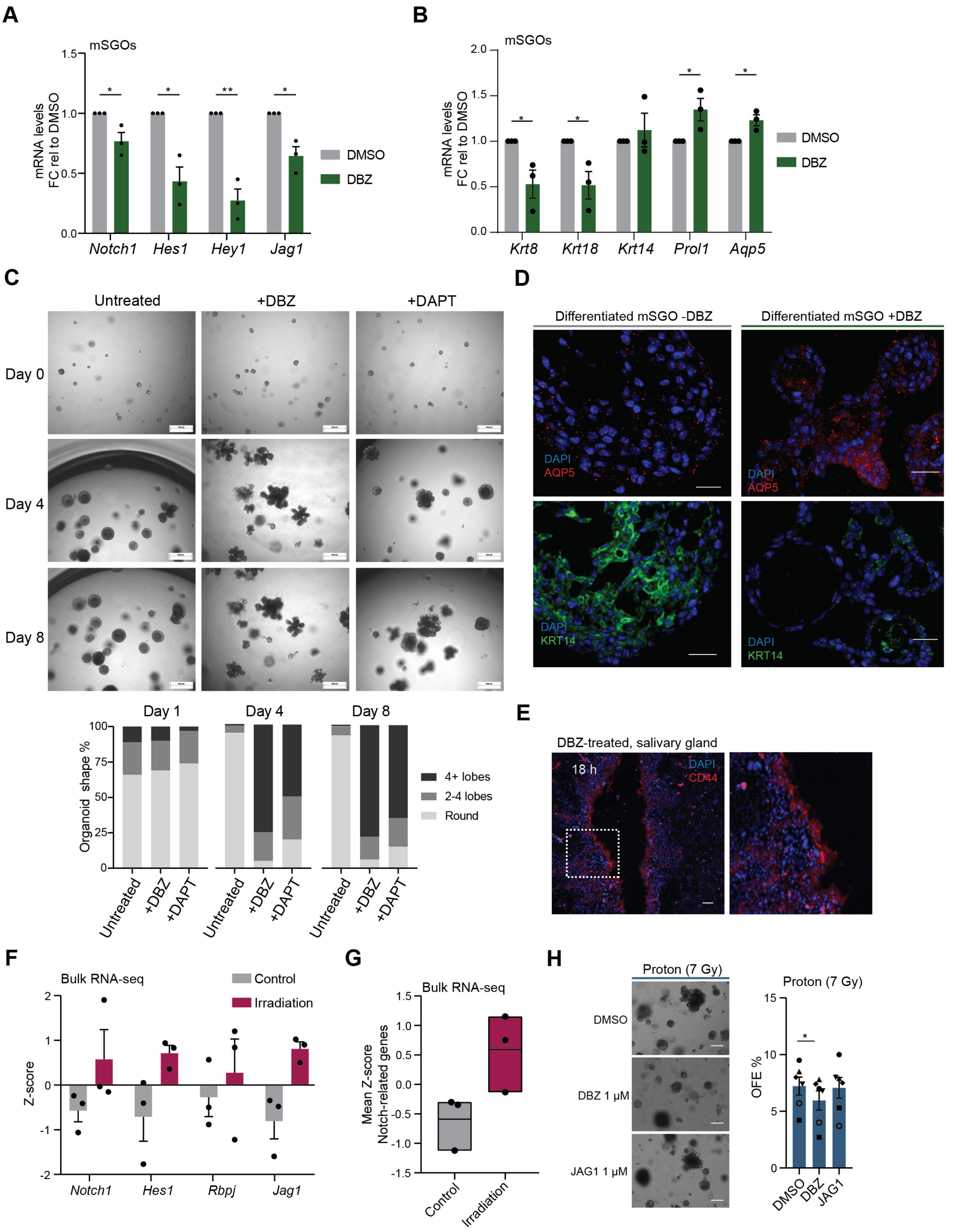

Appendix Figure S7. DBZ treatment in mSGOs

(**A**) rt-qPCR analysis of Notch-related genes in mSGOs after DMSO and DBZ treatment. Data is shown as FC relative to DMSO (means ± s.e.m; n = 3 animals/condition). Two-sided unpaired *t*-test (*Notch1* **p* = 0.046; *Hes1* **p* = 0.021; *Hey1* ***p* = 0.0034; *Jag1* **p* = 0.039). (**B**) rt-qPCR analysis of *Krt8* and *Krt18* (duct cells), *Krt14* (basal duct cells), *Prol1* and *Aqp5* (acinar cells) after DMSO and DBZ treatment. Data is shown as FC relative

to DMSO (means ± s.e.m; n = 3 animals/condition). Two-sided unpaired *t*-test (*Krt8* **p* = 0.037; *Krt18* **p* = 0.033; *Prol1* ***p* = 0.049; *Aqp5* **p* = 0.019). (**C**) Whole-well images of differentiated mSGOs after DBZ and DAPT treatment and quantification of round and lobular structures. (**D**) Representative images of immunofluorescence staining of differentiated mSGOs with or without DBZ treatment showing the expression of KRT14 and AQP5. Scale bar, 10 µm. (**E**) Representative images of immunofluorescence staining of salivary glands cells 18 h after wound generation and DBZ treatment showing the expression of CD44. Scale bar, 100 µm. (**F**) Expression of Notch-related genes extrapolated from the bulk RNA-seq analysis of control and photon-irradiated mSGOs. Data is shown as Z-score (n = 3 animals/condition). (**G**) Mean Z-score of the Notch-related genes shown in Appendix Fig. S7F. (**H**) Representative images and organoid quantification of proton-irradiated mSGOs after treatment with DMSO, DBZ and JAG1 (means ± s.e.m; n = 5 animals/condition). One-way ANOVA, post-hoc Tukey`s test (**p* = 0.045). Scale bar, 100 µm.

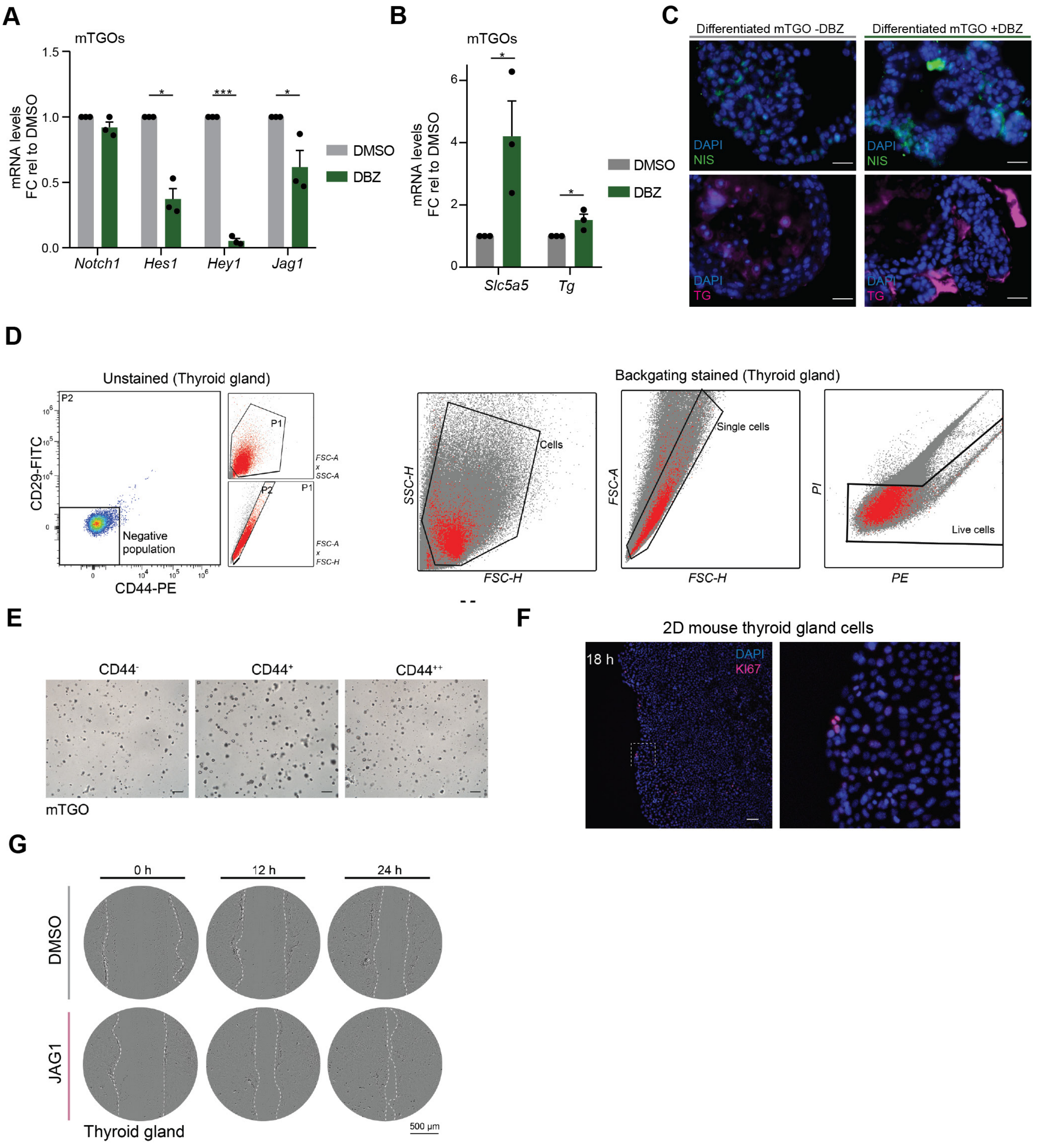

Appendix Figure S8. DBZ treatment in mTGOs

(**A**) rt-qPCR analysis of Notch-related genes in mTGOs after DMSO and DBZ treatment. Data is shown as FC relative to DMSO (means ± s.e.m; n = 3 animals/condition). Two-sided unpaired *t*-test (*Hes1* *p = 0.014; *Hey1* ***p = 0.00061; *Jag1* *p = 0.041). (**B**) rt-qPCR analysis of *Slc5a5* and *Tg* in mTGOs after DMSO and DBZ treatment. Data is shown as FC relative to DMSO (means ± s.e.m; n = 3 animals/condition). Two-sided unpaired *t*-test (*Slc5a5* *p = 0.048; *Tg* *p = 0.037). (**C**) Representative images of immunofluorescence staining of differentiated mTGOs with or without DBZ treatment showing the expression of NIS and TG. Scale bar, 20 μm. (**D**) Representative FACS plot of an unstained sample using the same gating strategy shown in Fig. 5E and backgating of FACS plot shown in Figure 5E. (**E**) Representative images of sorted thyroid cells after 1 week in culture (P2). Scale bar, 100 μm. (**F**) Representative immunofluorescence image of 2D thyroid gland cells under serum-starvation conditions, 18 hours after wound generation, showing KI67 expression. Scale bar, 100 μm.

(**G**) Representative images of thyroid gland cells after DMSO or JAG1 treatment at 0, 12 and 24 h after wound generation. Dotted line shows wound borders.

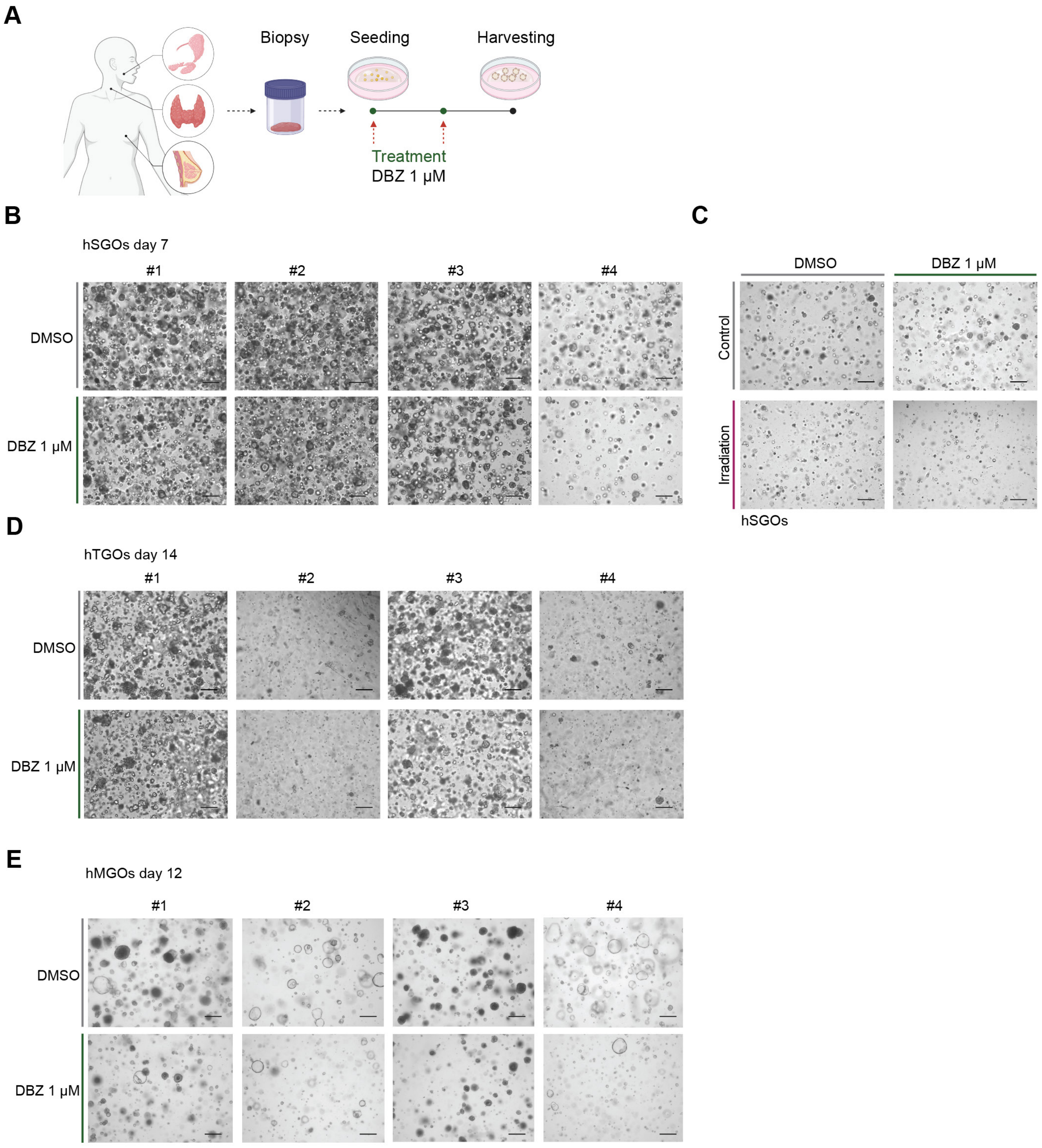

Appendix Figure S9. Patient-derived glandular organoids

(**A**) Schematic representation of DBZ treatment in hSGOs, hTGOs and hMGOs. (**B**) Representative images of hSGOs after DMSO and DBZ treatment. (**C**) Representative images of control and photon-irradiated hSGOs after treatment with DMSO, DBZ or JAG1. (**D**) Representative images of hTGOs after DMSO and DBZ treatment. (**E**) Representative images of hMGOs after DMSO and DBZ treatment. In all images numbers indicate different patients. Scale bar, 100 µm.

Gene	Forward Primer 5`- 3`	Reverse Primer 5` - 3`
Ywhaz	TTACTTGGCCGAGGTTGCT	TGCTGTGACTGGTCCACAAT
Notch1	AGCAAGAAGAAGCGGGAGAGC	TGTCGTCCATCAGAGCACCATC
Hes1	CACTGGAAGGTGACACTGCG	GAGAGGCTGCCAAGGTTTTTG
Jag1	ACACAGGGATTGCCCACTTC	AGCCAAAGCCATAGTAGTGGTCA
Hey1	CCCAAACTCCGATAGTCCATAGCC	GCCGACGAGACCGATCAATAAC
Nkx2-1	CGCCTTACCAGGACACCAT	CCCATGCCACTCATATTCAT
Slc5a5	TCCACAGGAATCATCTGCACC	CCACGGCCTTCATACCACC
Tpo	ACAGTCACAGTTCTCCACGGATG	ATCTCTATTGTTGCACGCCCC
Tg	AGGACCCGTGTGGTAGG	CTGACCCAGAGAATGGCAGT

Appendix Table S1. Primer sequences (mouse)
