# Supplementary figures and images for: Notch signaling is a driver of glandular stem cell activity and regenerative migration after damage
Source: EMBO J. 2025 Nov 5;45(2):374–93. doi: 10.1038/s44318-025-00607-w (PMC12811363; doi:10.1038/s44318-025-00607-w)

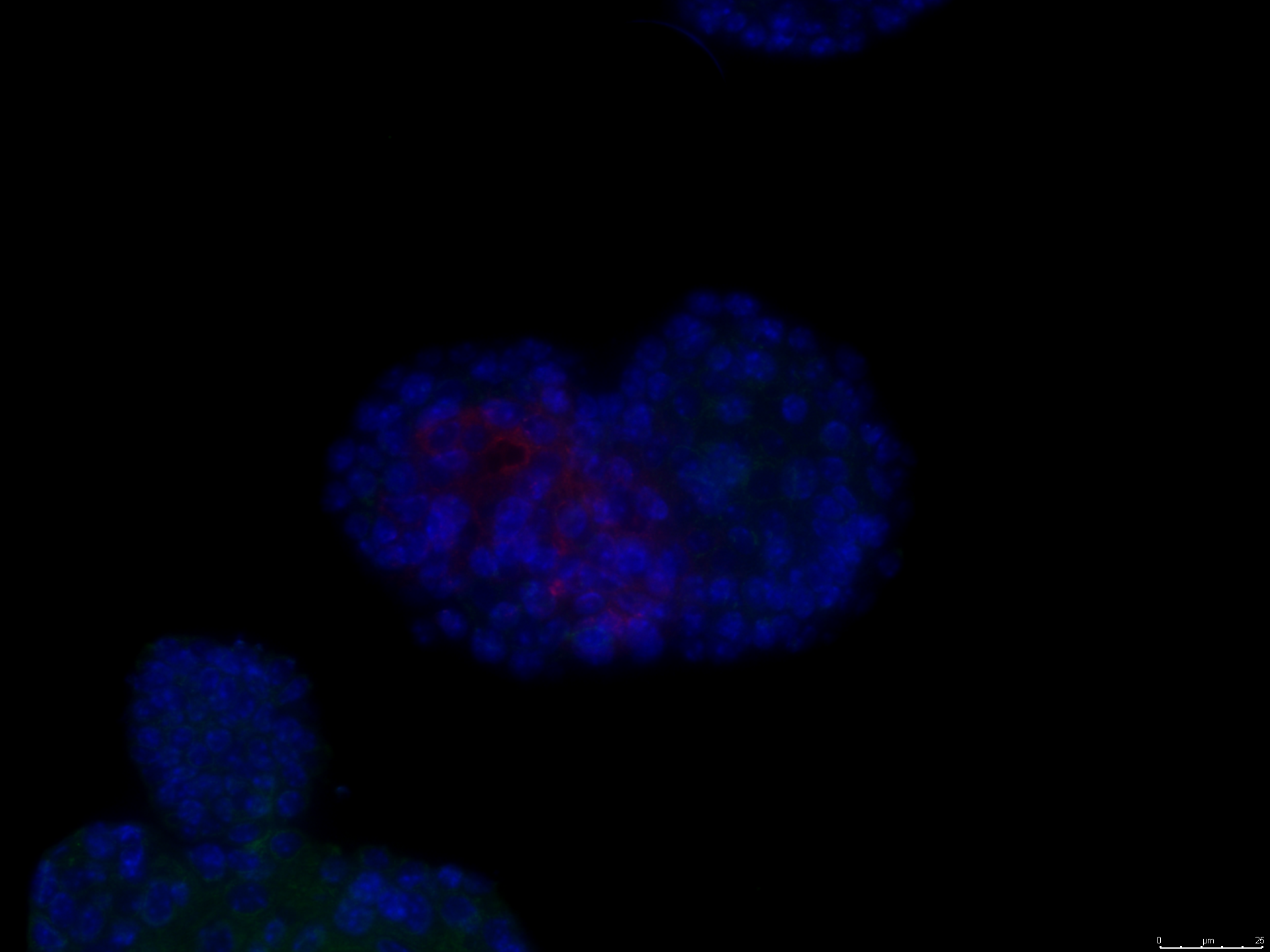

Supplement: Supplementary file 5 — Source data Fig. 1 [file 44318_2025_607_MOESM5_ESM.zip › Figure 1/1K/Day11_DAPI_KRT14_KRT8_overlay.tif]

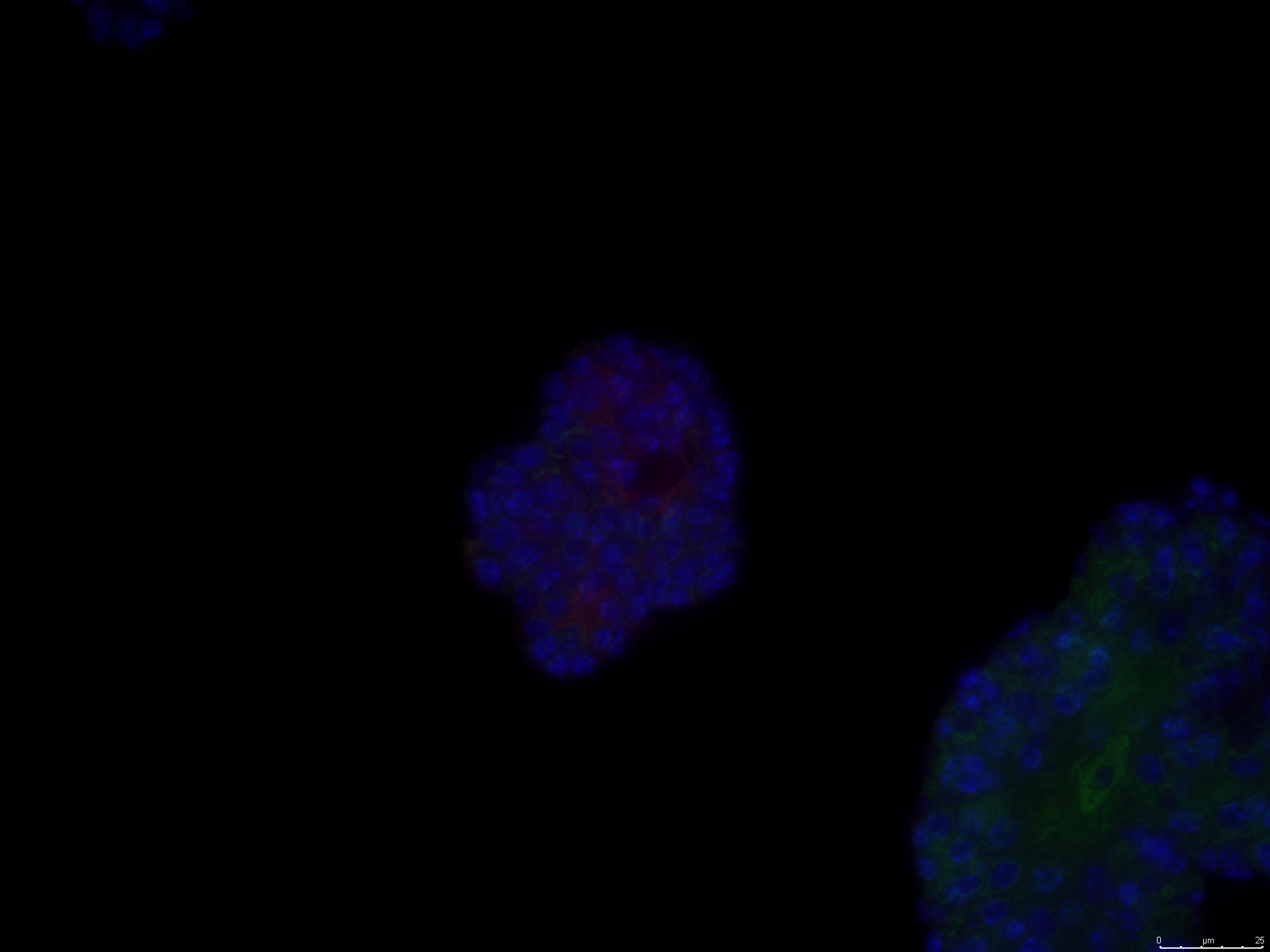

Supplement: Supplementary file 5 — Source data Fig. 1 [file 44318_2025_607_MOESM5_ESM.zip › Figure 1/1K/Day7_DAPI_KRT14_KRT8_overlay.tif]

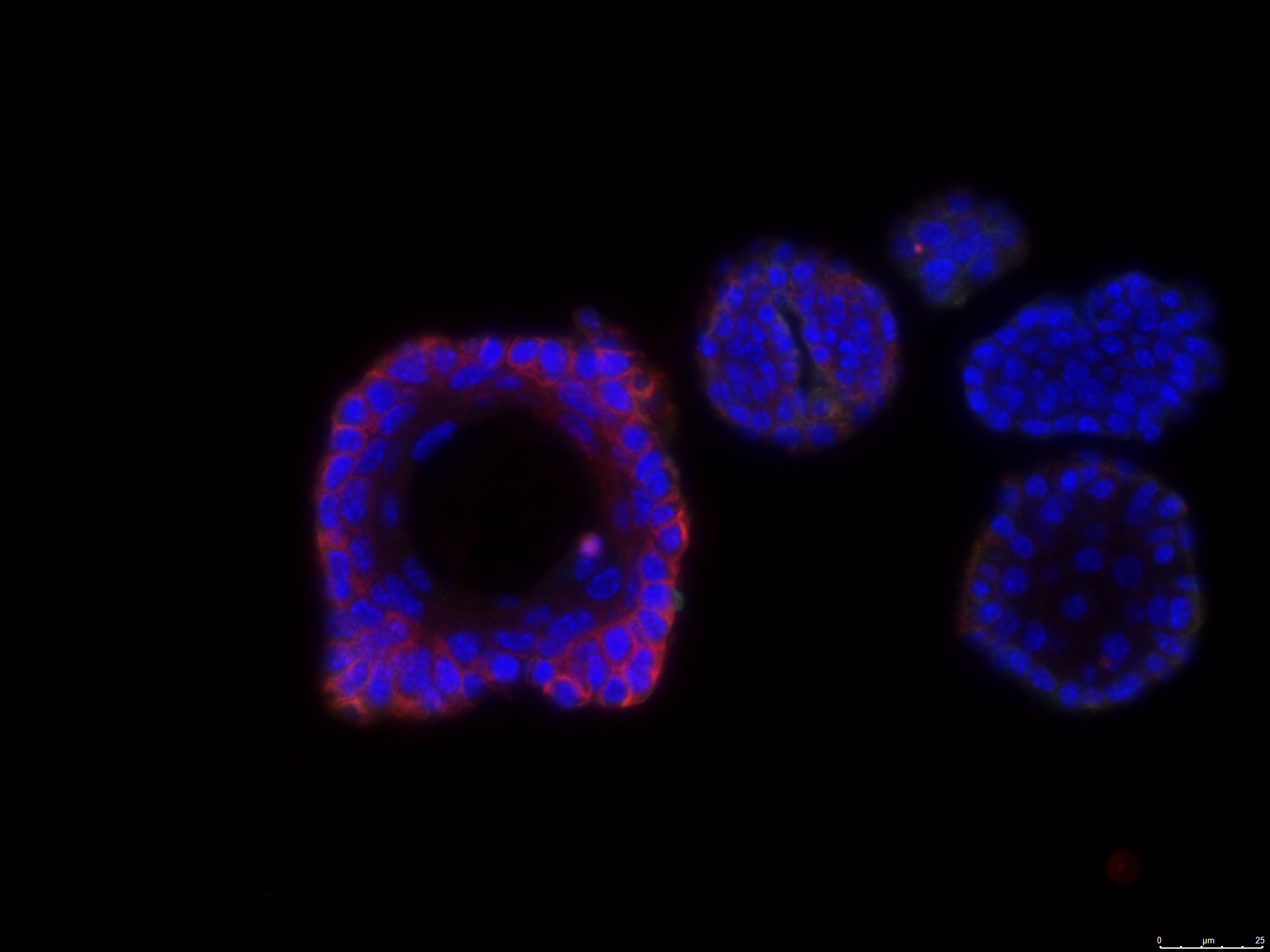

Supplement: Supplementary file 5 — Source data Fig. 1 [file 44318_2025_607_MOESM5_ESM.zip › Figure 1/1L/Day7_DAPI_CD44_CD29_overlay.tif]

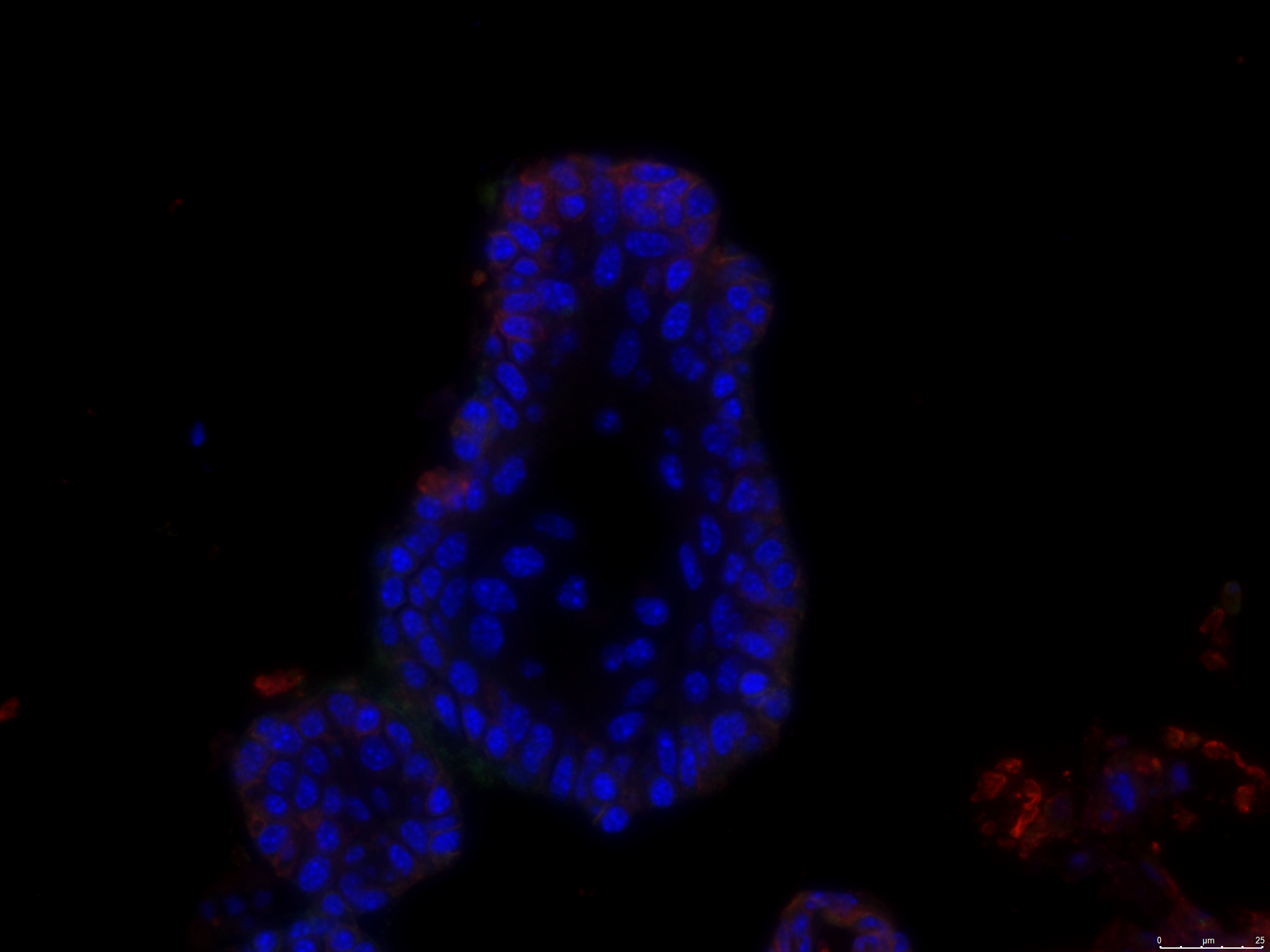

Supplement: Supplementary file 5 — Source data Fig. 1 [file 44318_2025_607_MOESM5_ESM.zip › Figure 1/1L/Day11_DAPI_CD44_CD29_overlay.tif]

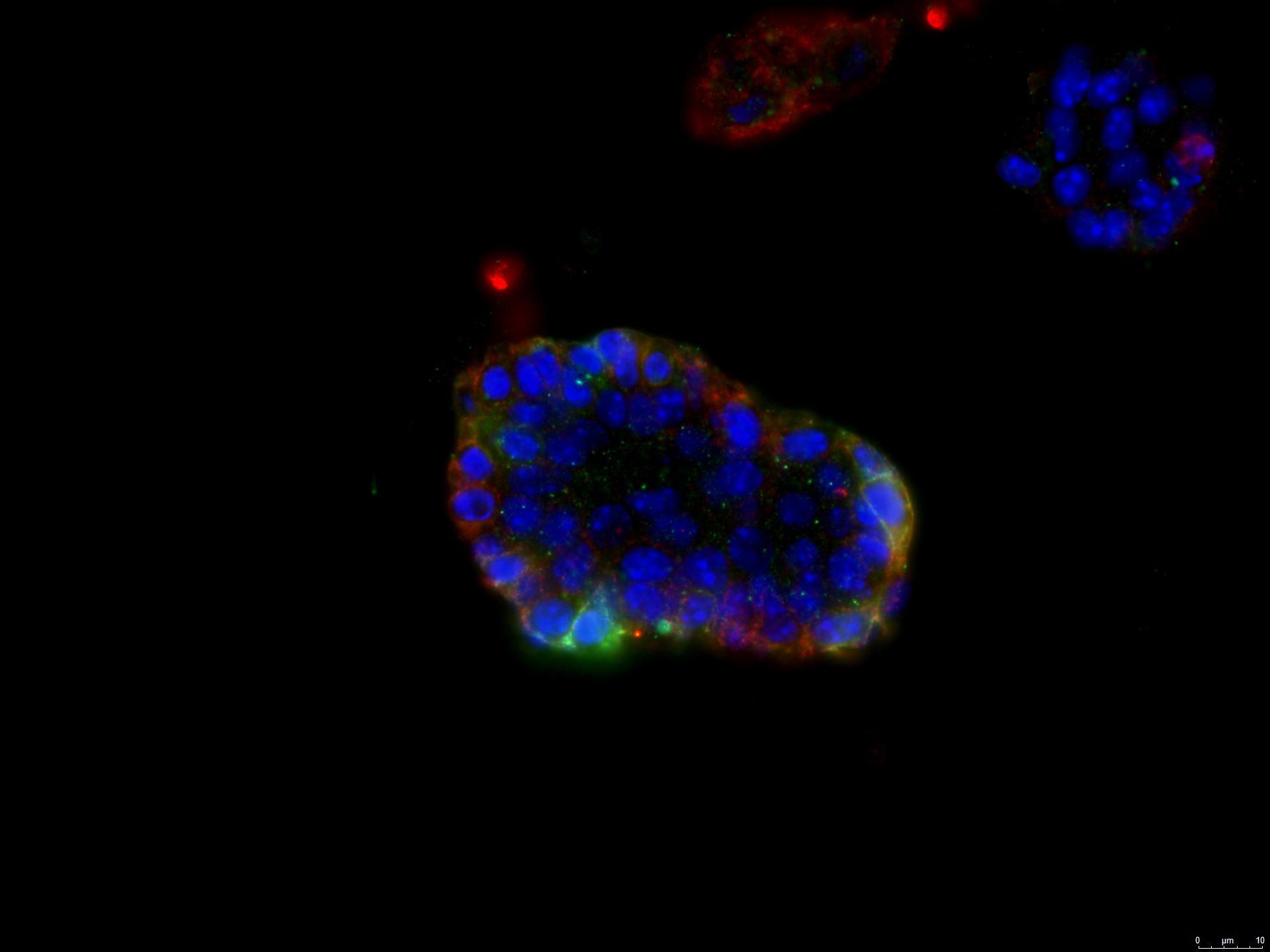

Supplement: Supplementary file 5 — Source data Fig. 1 [file 44318_2025_607_MOESM5_ESM.zip › Figure 1/1M/Day7_DAPI_CD44_AQP5_overlay.tif]

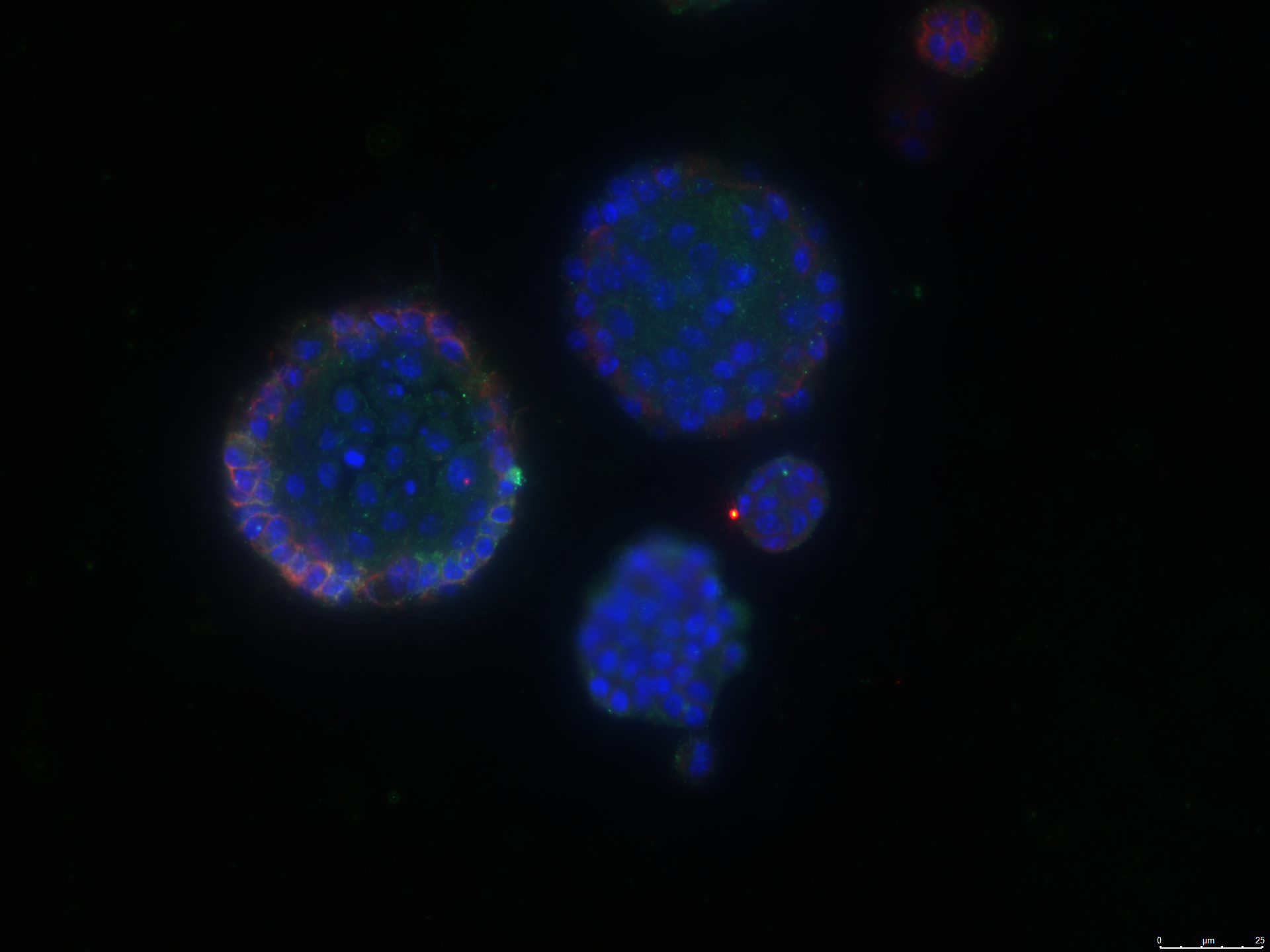

Supplement: Supplementary file 5 — Source data Fig. 1 [file 44318_2025_607_MOESM5_ESM.zip › Figure 1/1M/Day11_DAPI_CD44_AQP5_overlay.tif]

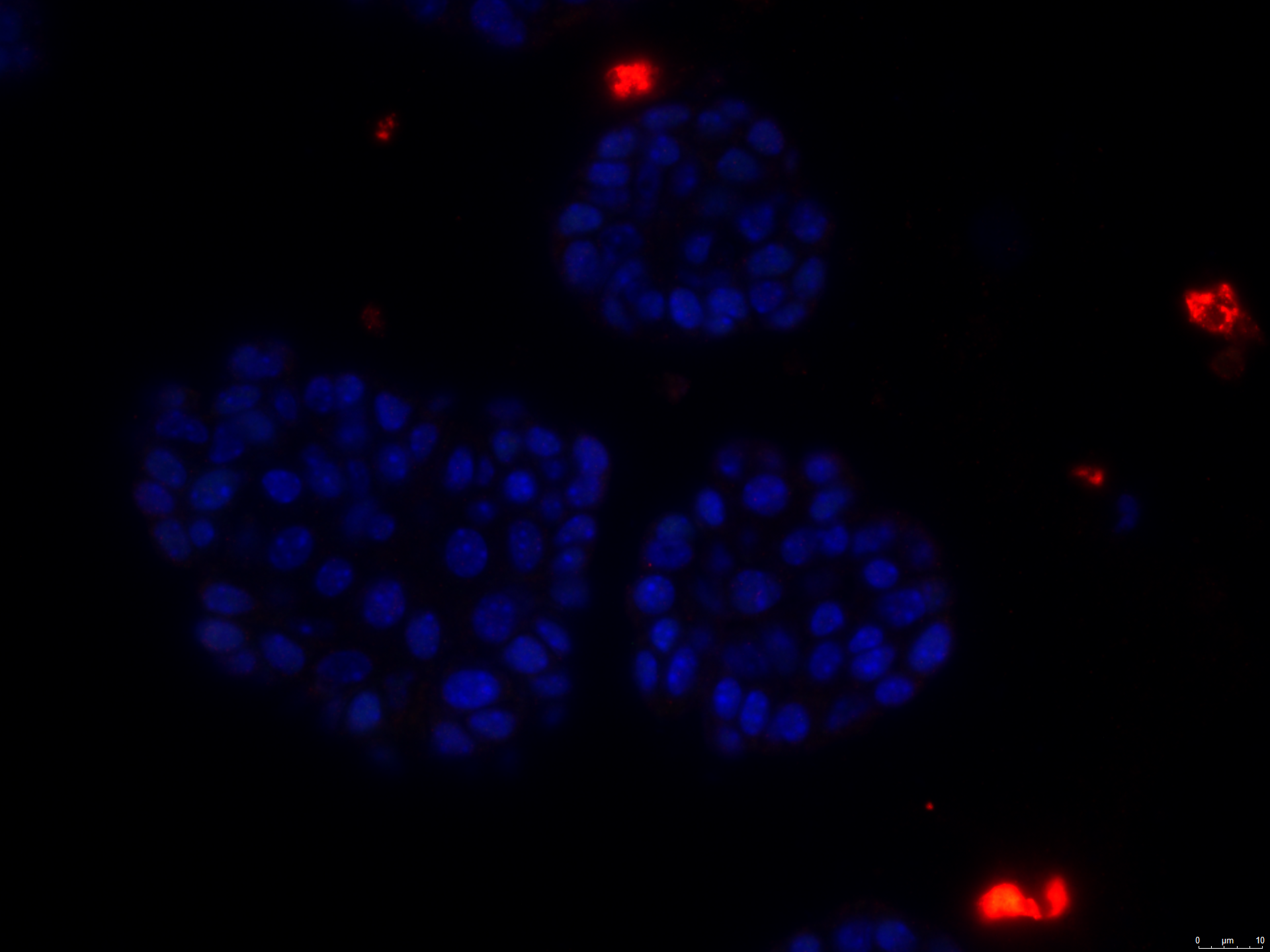

Supplement: Supplementary file 5 — Source data Fig. 1 [file 44318_2025_607_MOESM5_ESM.zip › Figure 1/1N/Day11_DAPI_CD164_SOX9_overlay.tif]

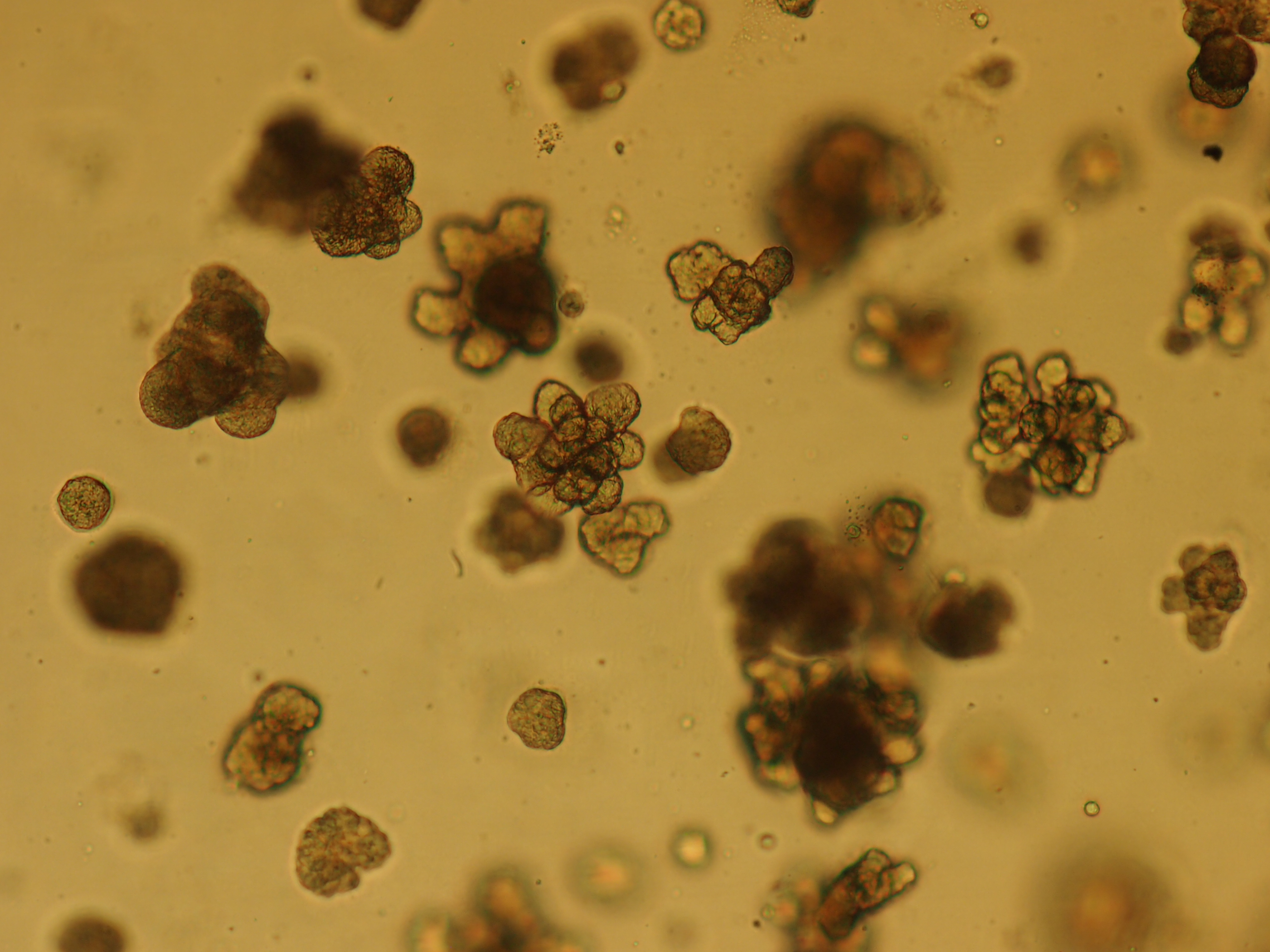

Supplement: Supplementary file 5 — Source data Fig. 1 [file 44318_2025_607_MOESM5_ESM.zip › Figure 1/1C/mSGO day 11.JPG]

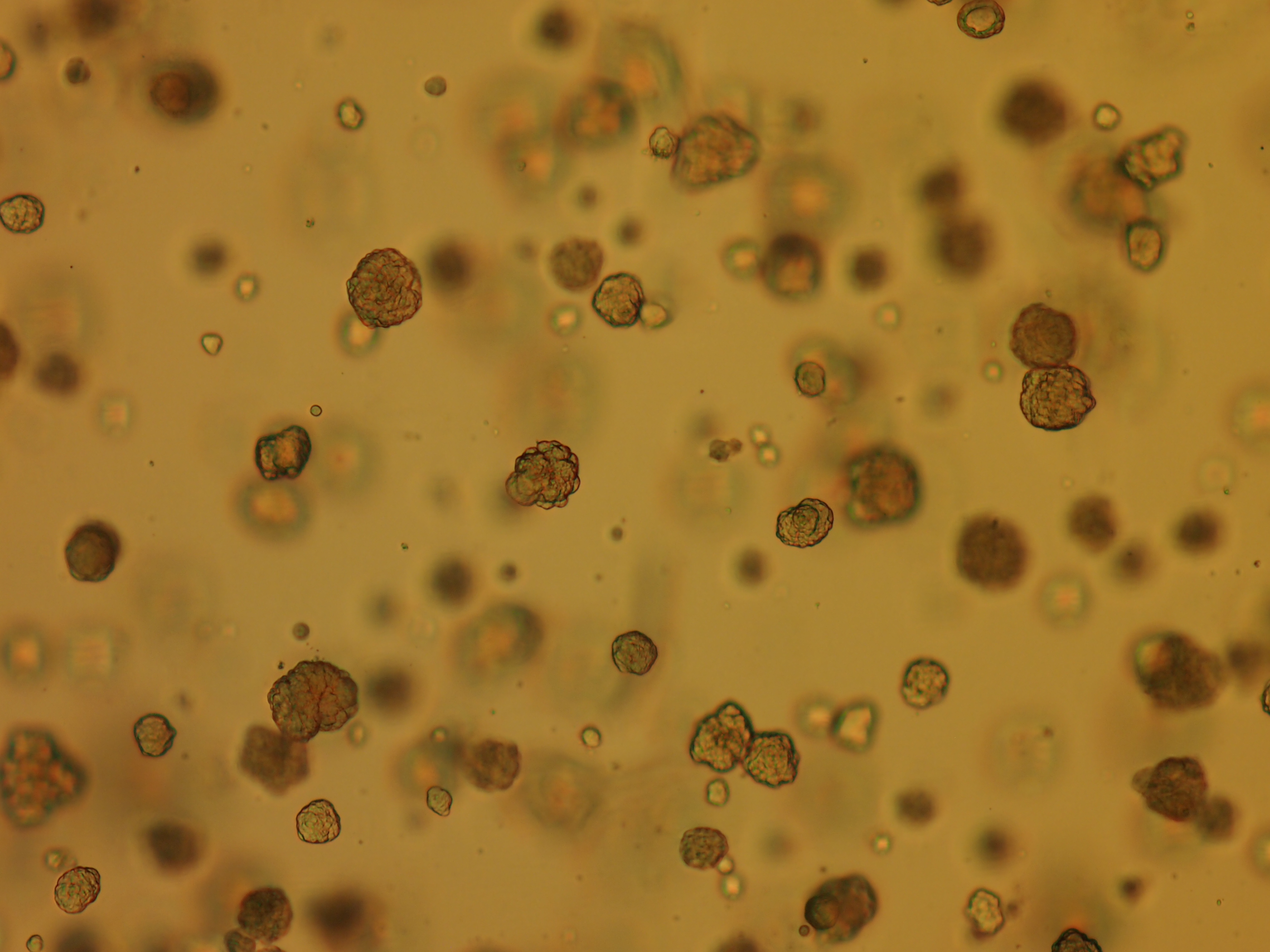

Supplement: Supplementary file 5 — Source data Fig. 1 [file 44318_2025_607_MOESM5_ESM.zip › Figure 1/1B/mSGO day 7.JPG]

CD29low\_CD24low

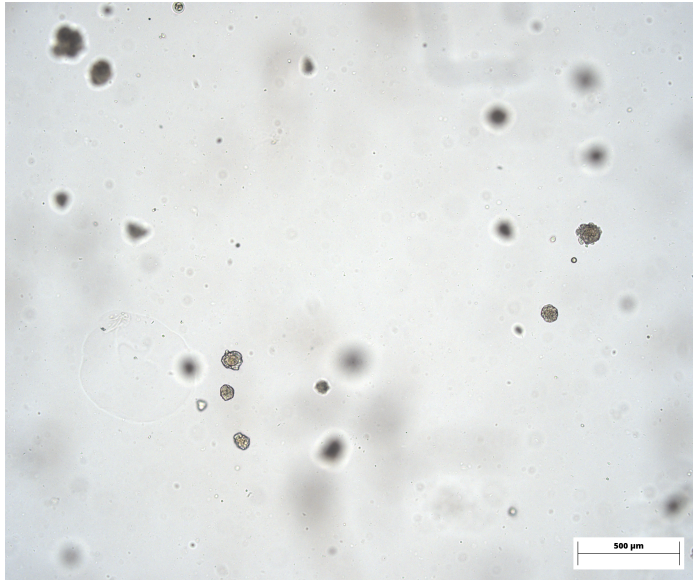

CD29low\_CD24high

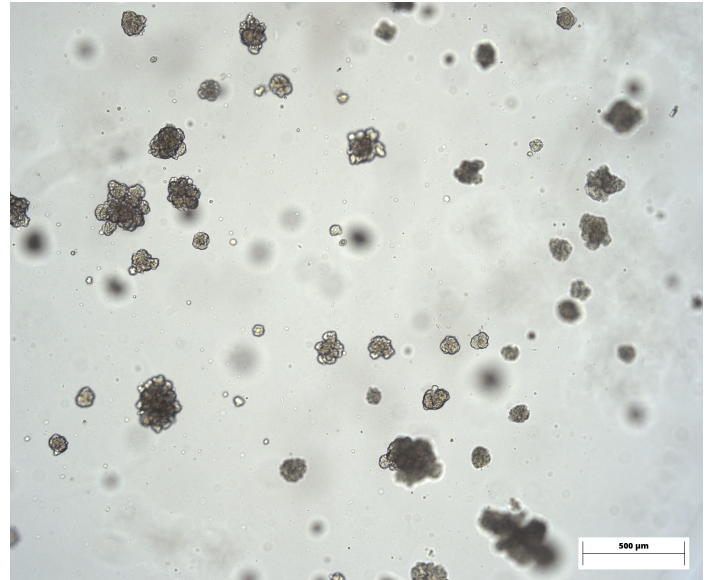

CD29high\_CD24low

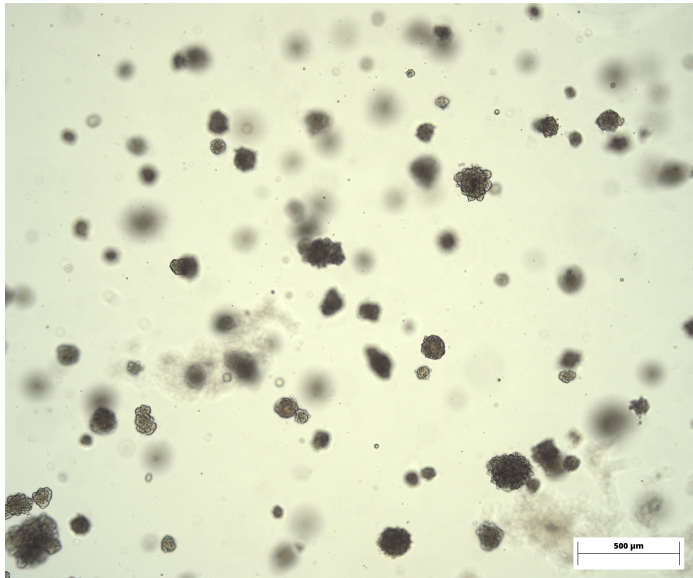

CD29high\_CD24high

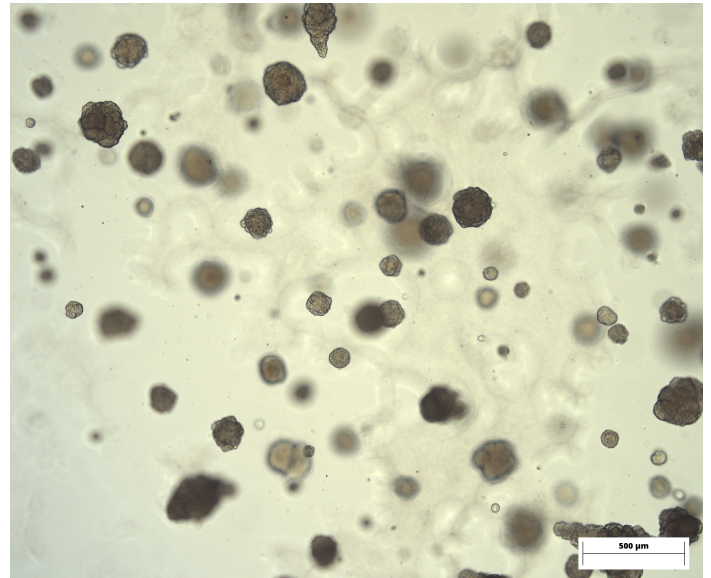

Supplement: Supplementary file 6 — Source data Fig. 2 [file 44318_2025_607_MOESM6_ESM.zip › Figure 2/Figure 2/2H/mSGO after FACS.pdf]

DAY 7 - 0 h

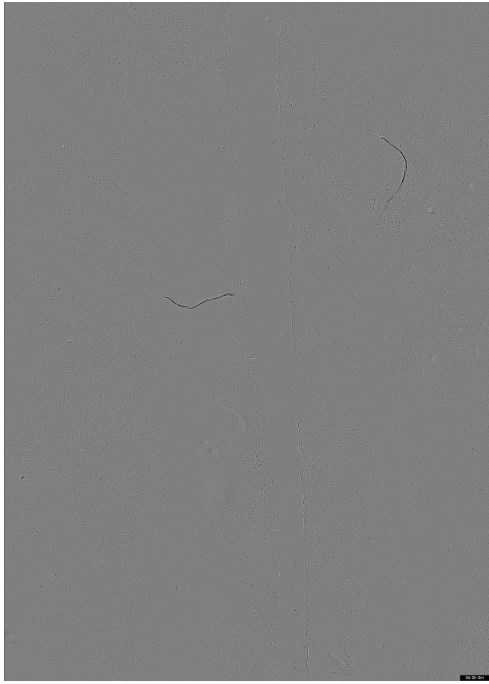

DAY 7 - 12 h

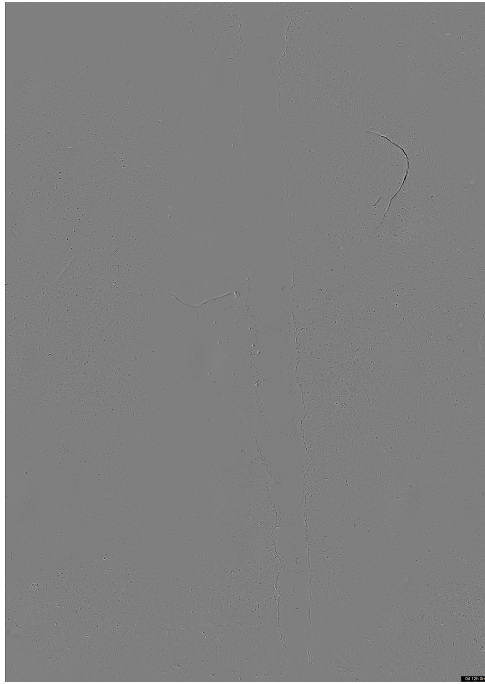

DAY 7 - 24 h

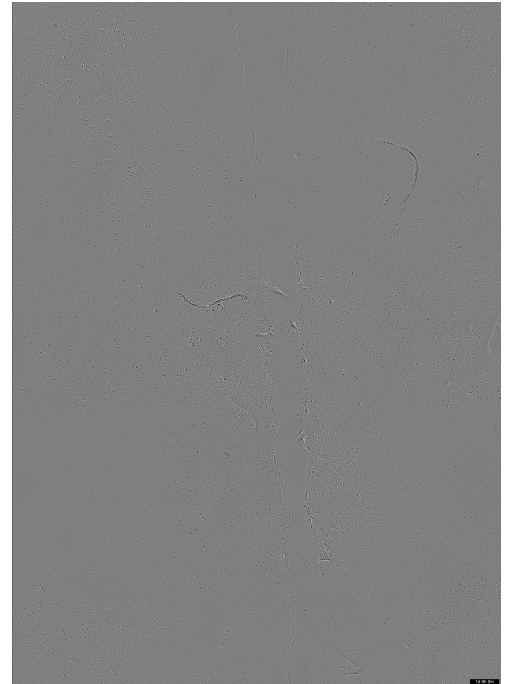

DAY 11 - 0 h

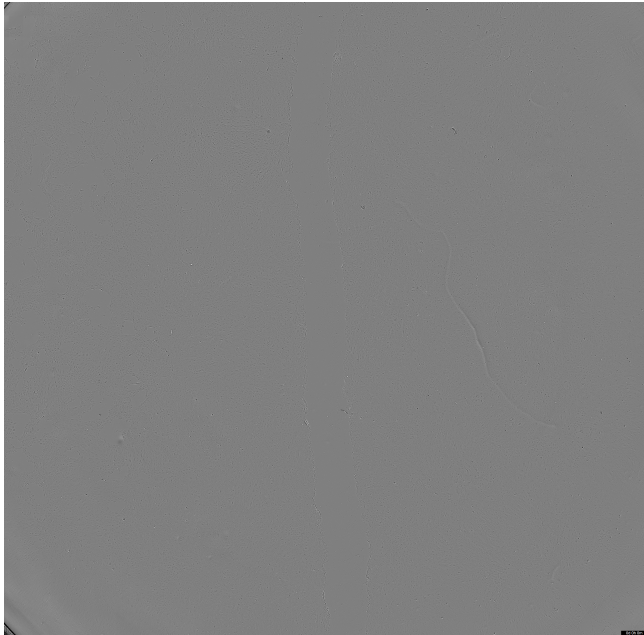

DAY 11 - 12 h

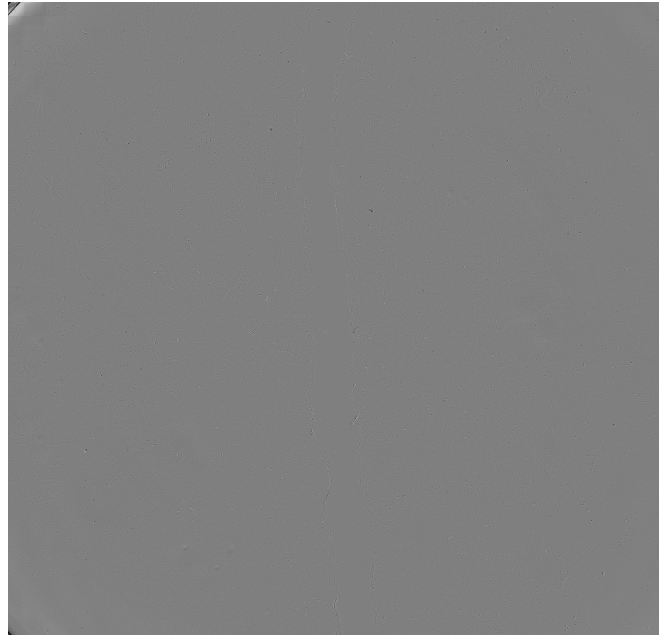

DAY 11 - 24 h

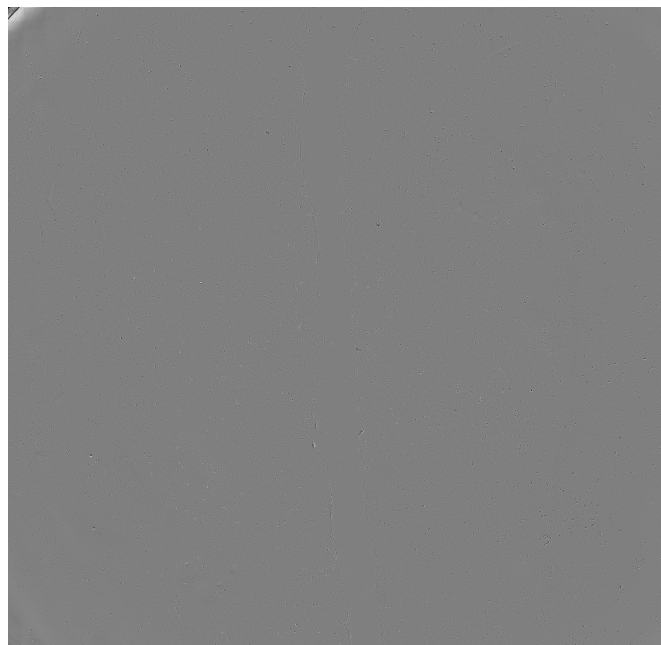

Supplement: Supplementary file 6 — Source data Fig. 2 [file 44318_2025_607_MOESM6_ESM.zip › Figure 2/Figure 2/2M/Microscopy scratch.pdf]

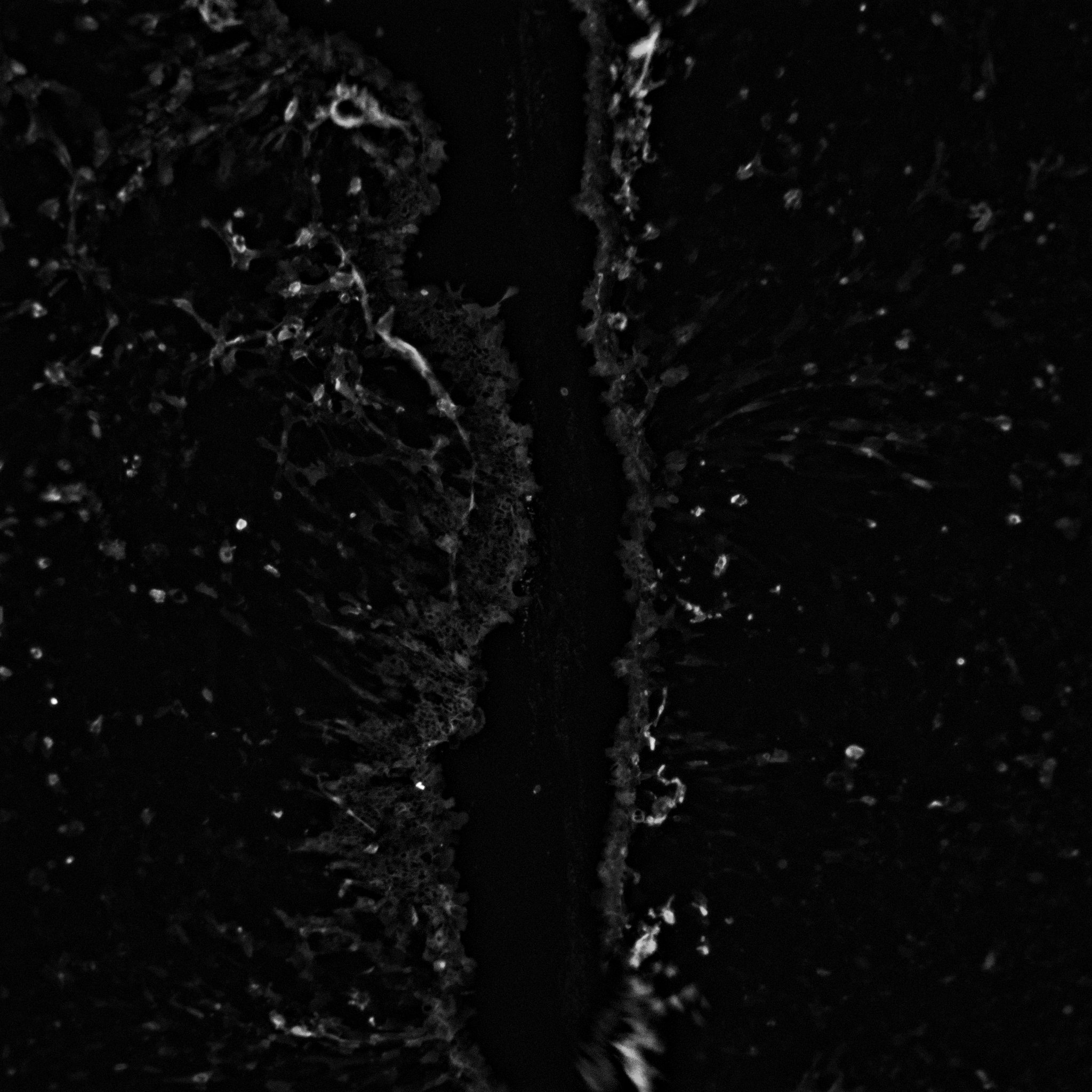

Supplement: Supplementary file 6 — Source data Fig. 2 [file 44318_2025_607_MOESM6_ESM.zip › Figure 2/Figure 2/2O/Day7_CD44.tif]

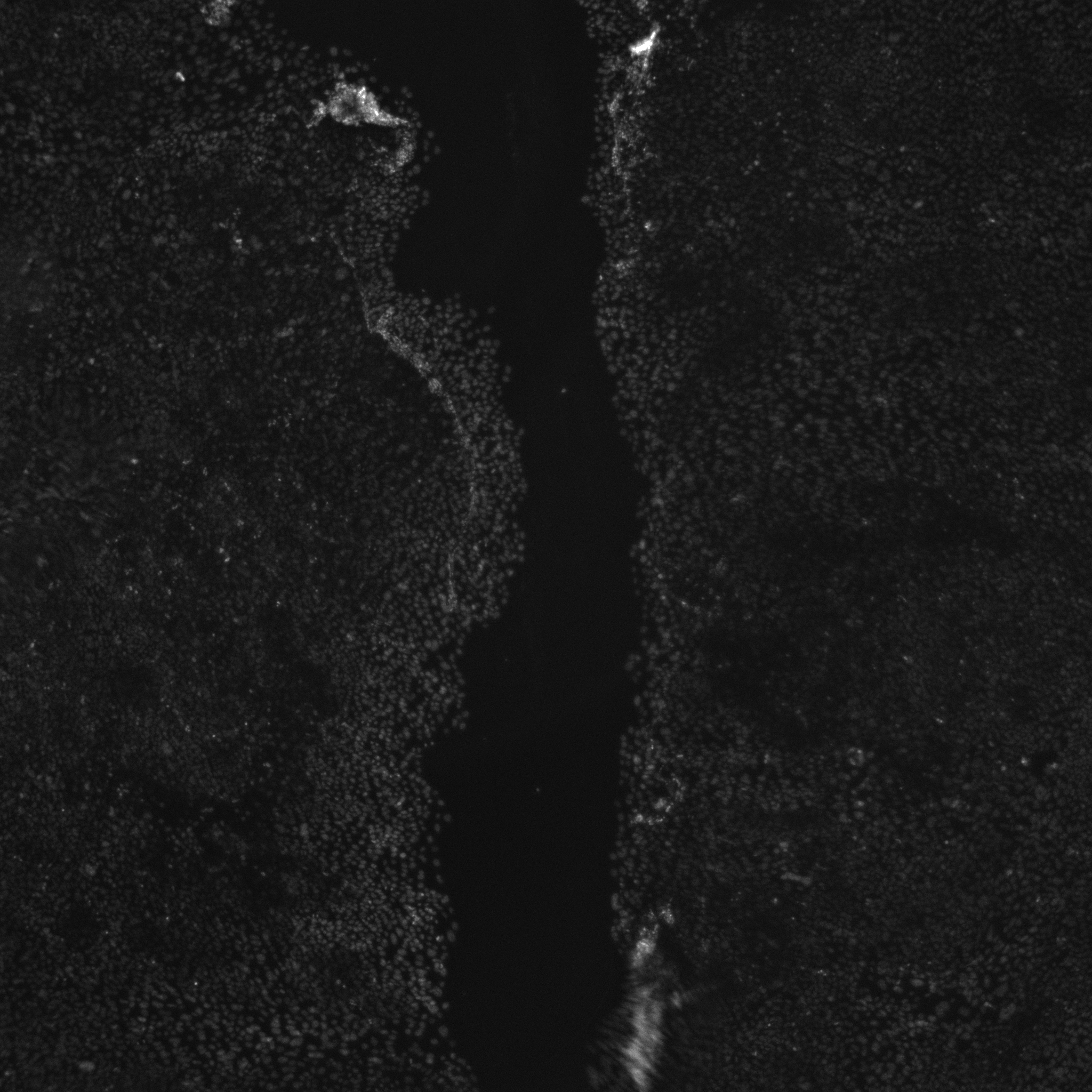

Supplement: Supplementary file 6 — Source data Fig. 2 [file 44318_2025_607_MOESM6_ESM.zip › Figure 2/Figure 2/2O/Day7_DAPI.tif]

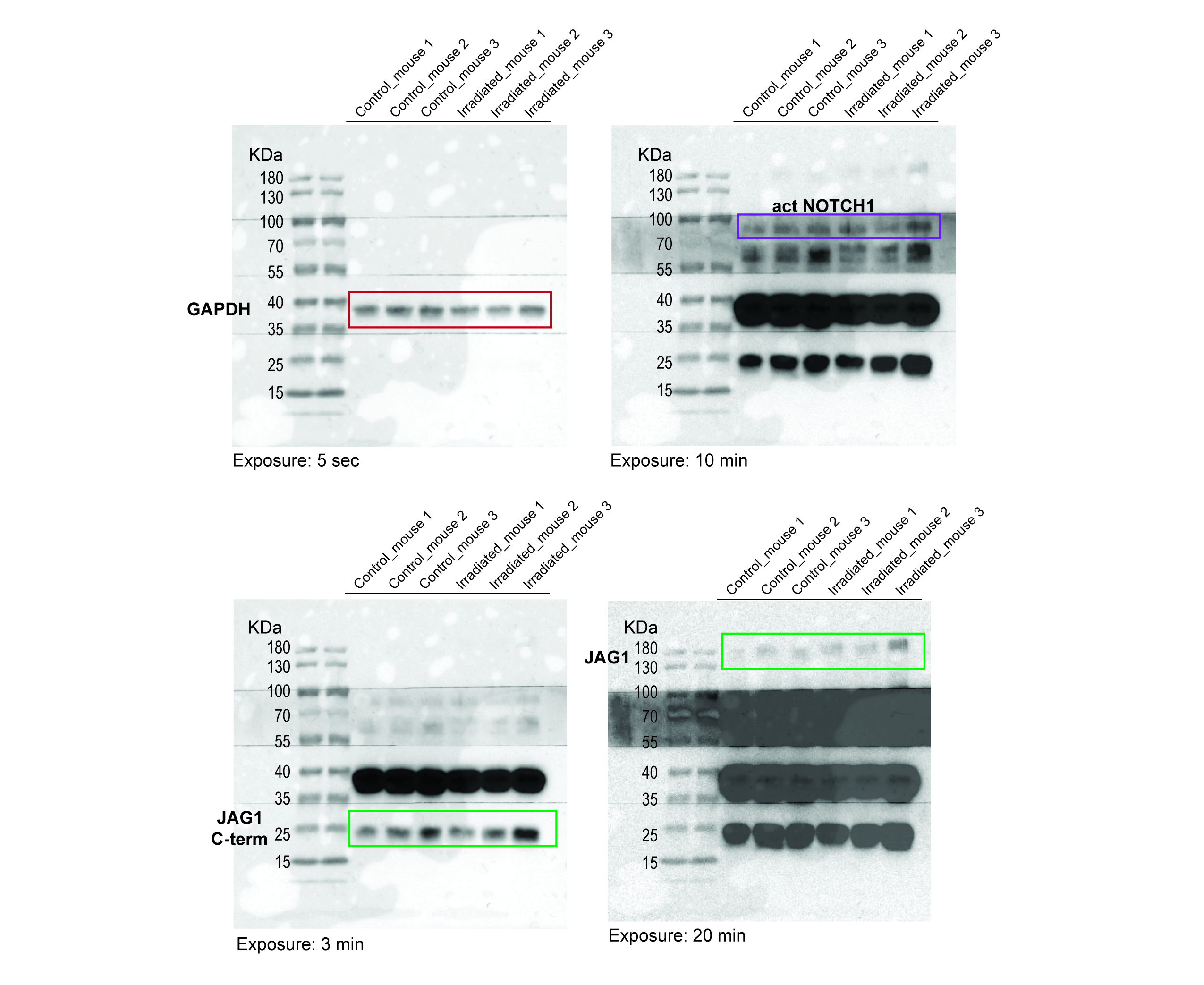

Supplement: Supplementary file 7 — Source data Fig. 3 [file 44318_2025_607_MOESM7_ESM.zip › Figure 3/3J/Western blot.tif]

DMSO

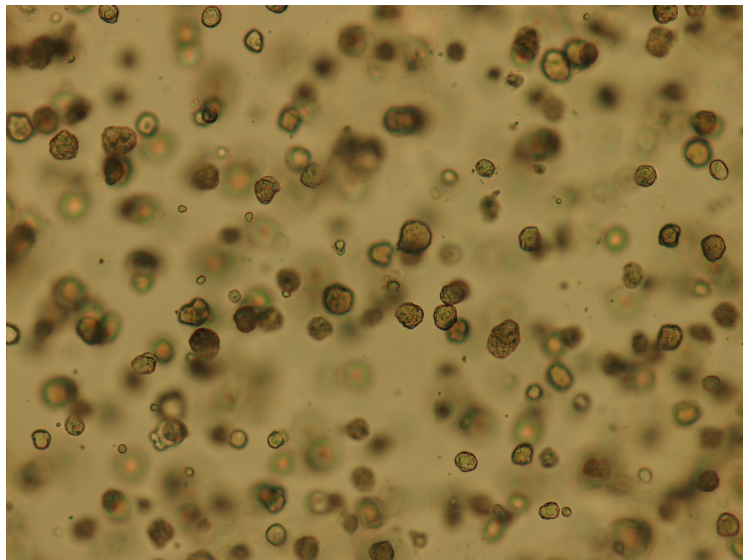

DBZ

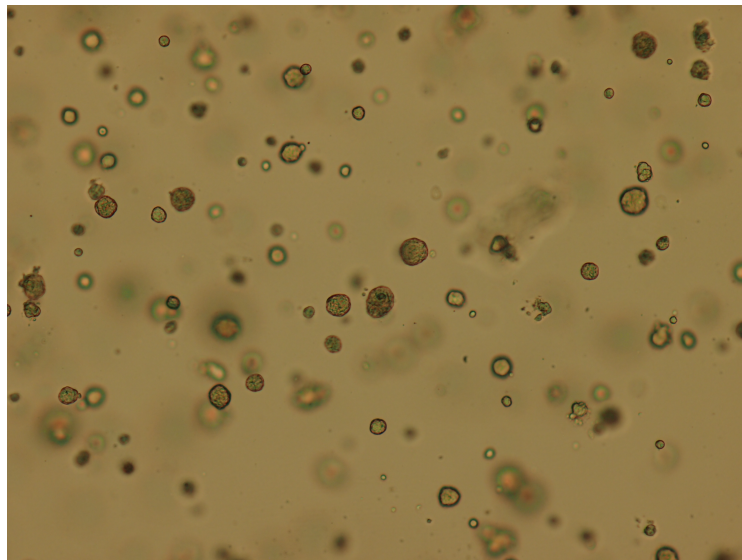

Supplement: Supplementary file 8 — Source data Fig. 4 [file 44318_2025_607_MOESM8_ESM.zip › Figure 4/4B/Microscopy mSGO_DMSO and DBZ.pdf]

Day 0 +DBZ

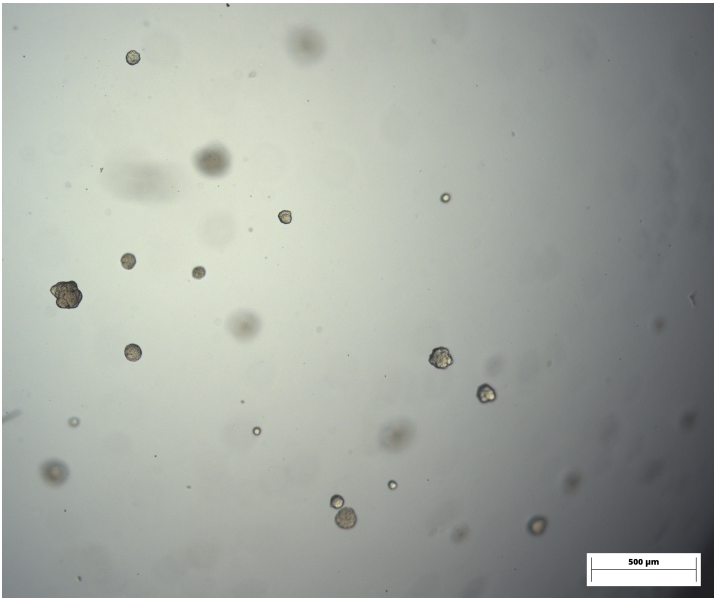

Day 0 -DBZ

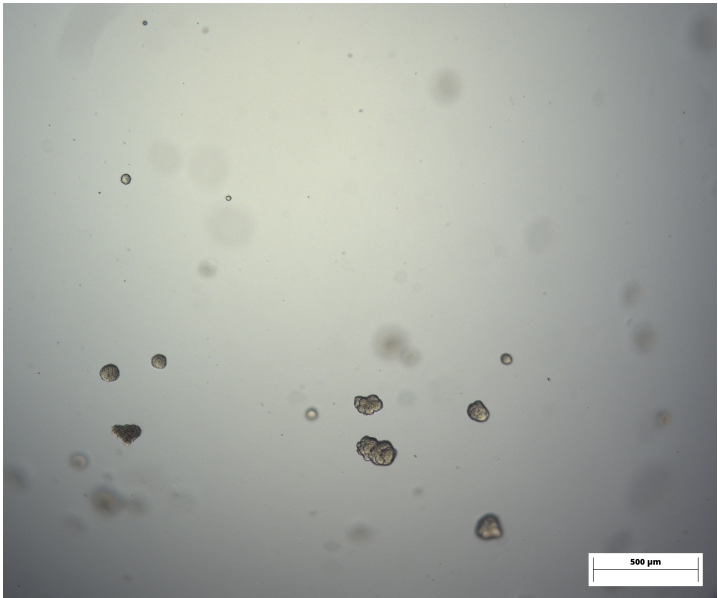

Day 4 +DBZ

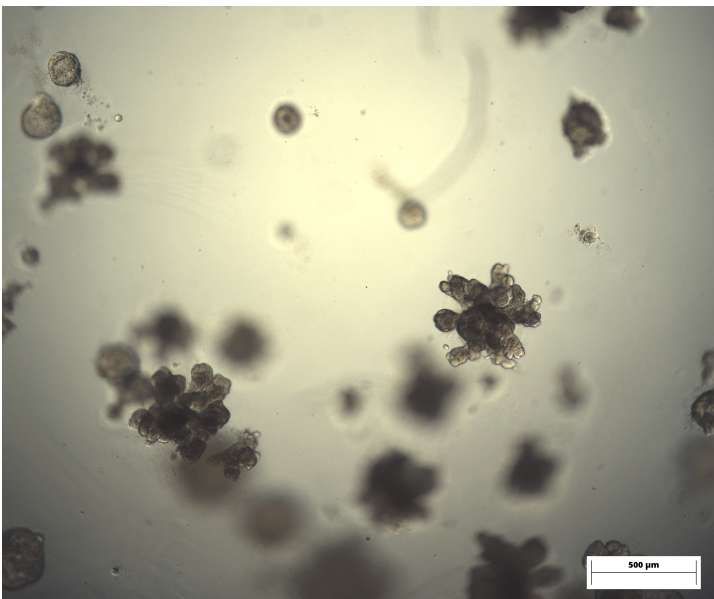

Day 4 -DBZ

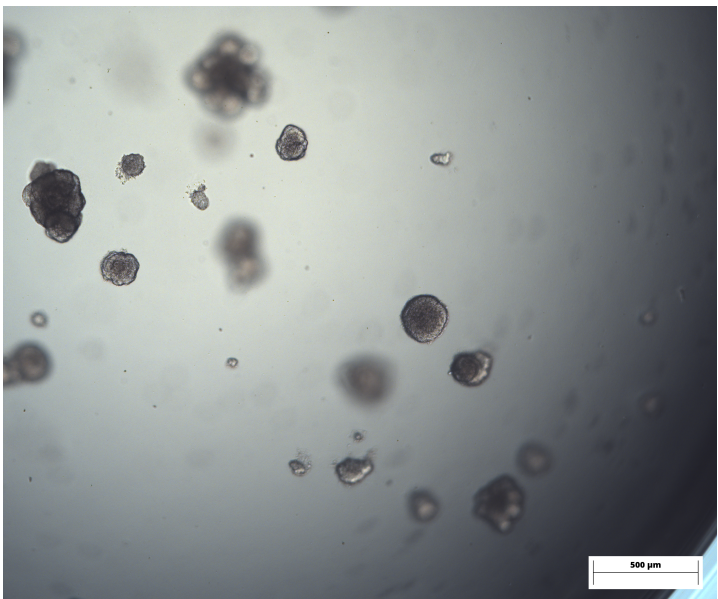

Day 8 +DBZ

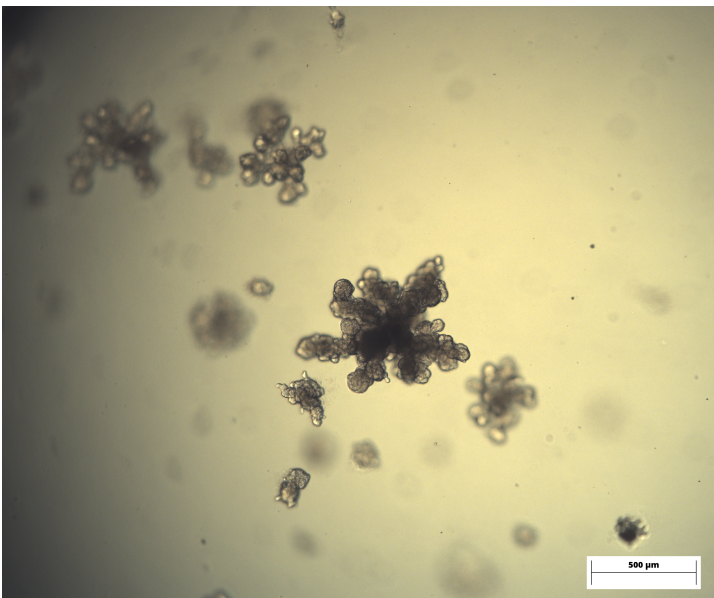

Day 8 -DBZ

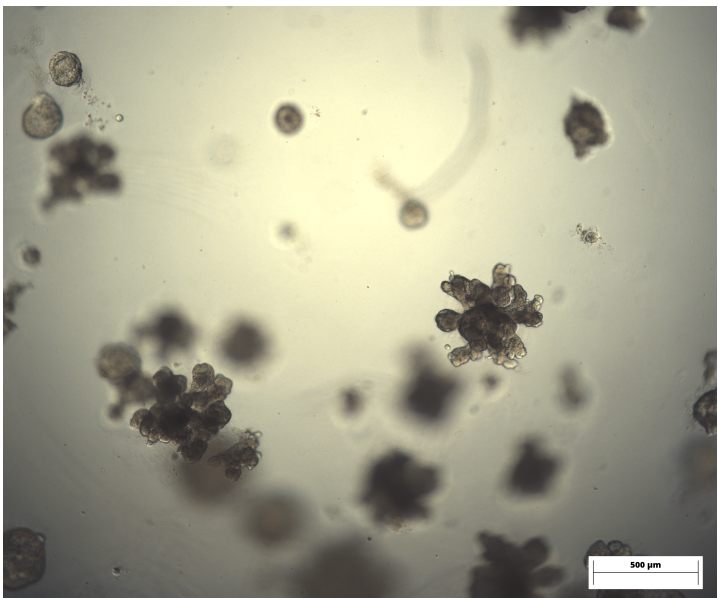

Supplement: Supplementary file 8 — Source data Fig. 4 [file 44318_2025_607_MOESM8_ESM.zip › Figure 4/4E/Microscopy differentiated mSGO_DMSO and DBZ.pdf]

-DBZ

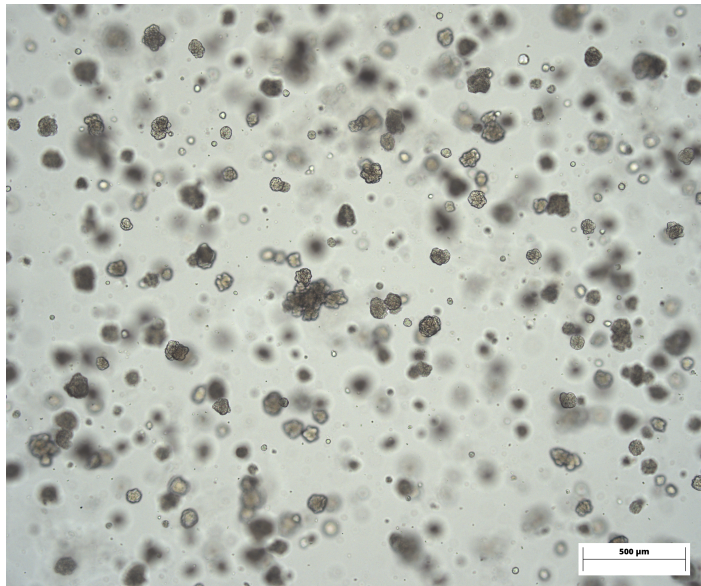

+DBZ

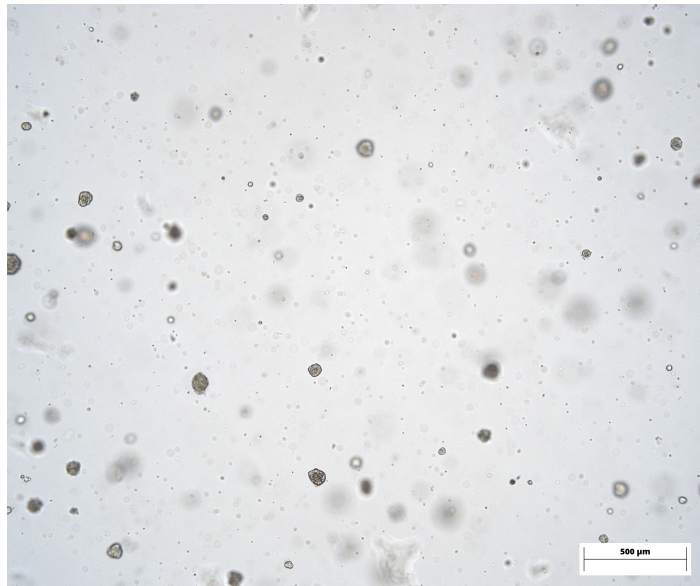

Supplement: Supplementary file 8 — Source data Fig. 4 [file 44318_2025_607_MOESM8_ESM.zip › Figure 4/4F/Microscopy mSGO after self-renewal.pdf]

DMSO - 0h

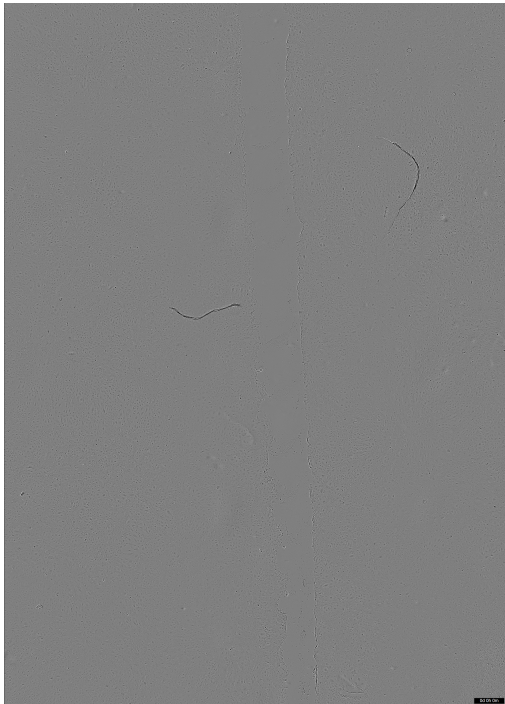

DMSO - 12h

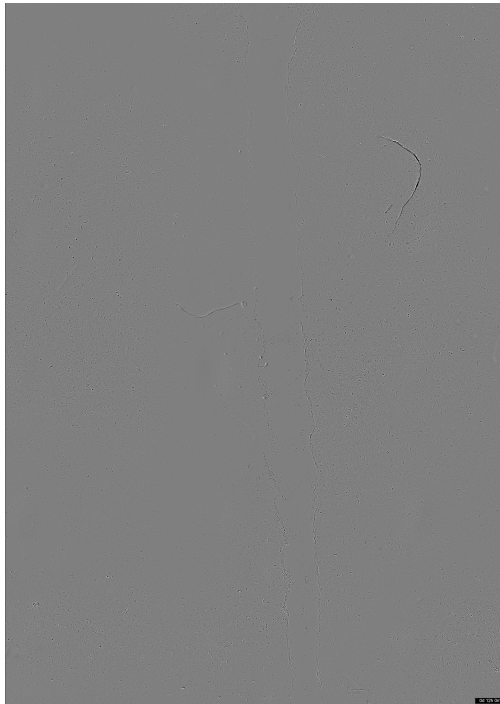

DMSO - 24h

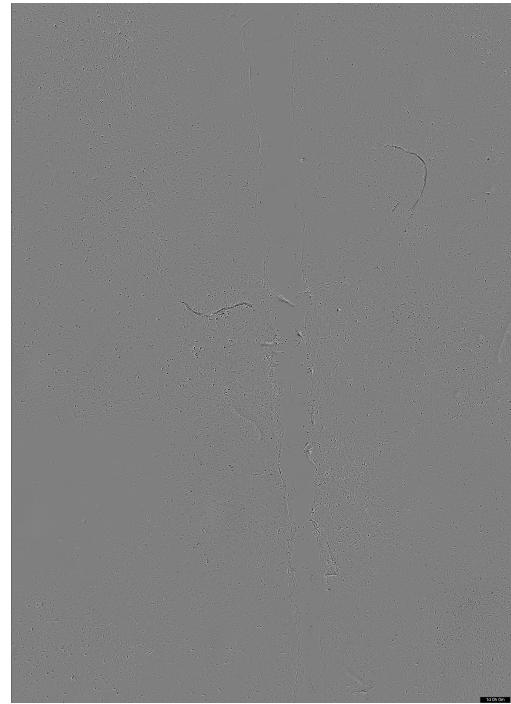

DBZ - 0h

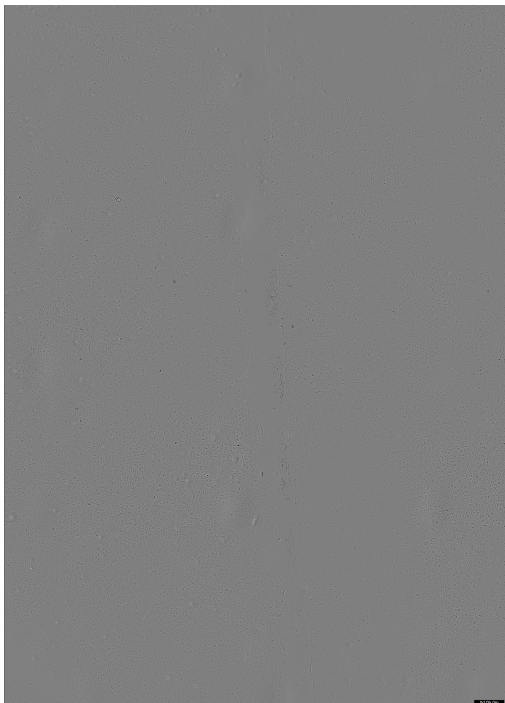

DBZ - 12h

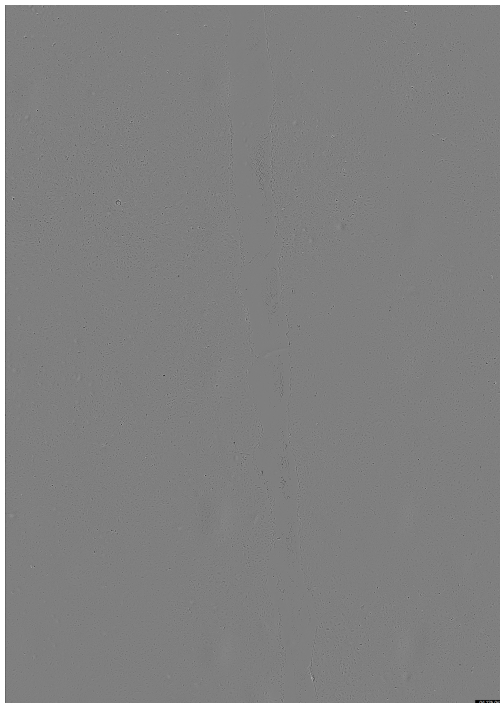

DBZ - 24h

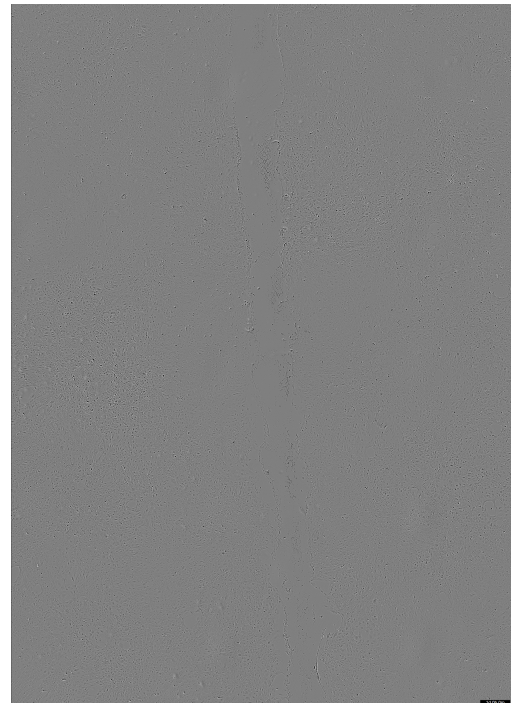

Supplement: Supplementary file 8 — Source data Fig. 4 [file 44318_2025_607_MOESM8_ESM.zip › Figure 4/4H/Microscopy scratch mSGO_DMSO and DBZ.pdf]

Control DBZ

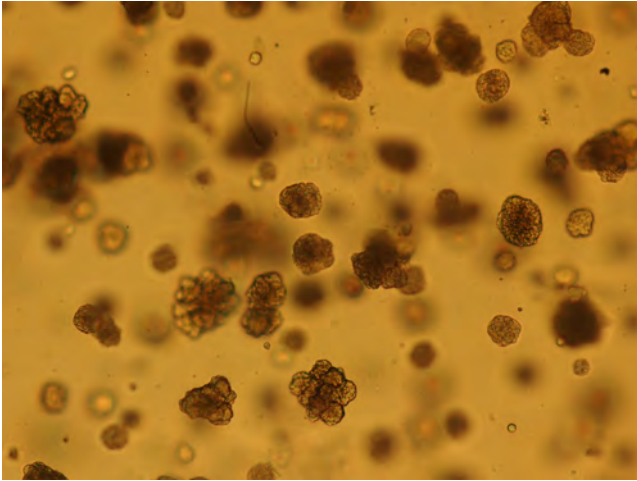

Control DMSO

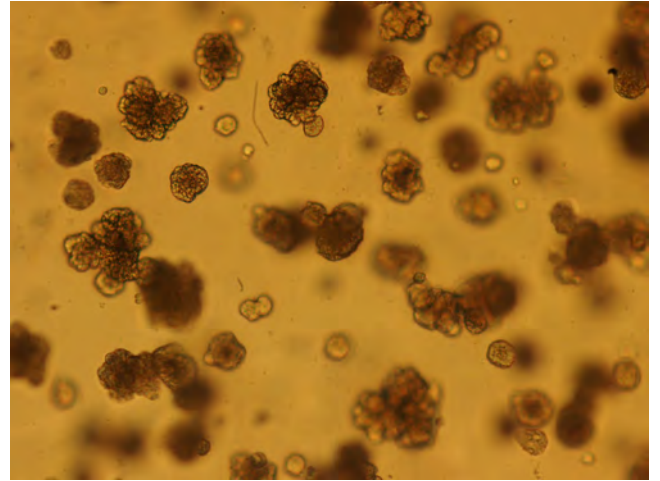

Control JAG1

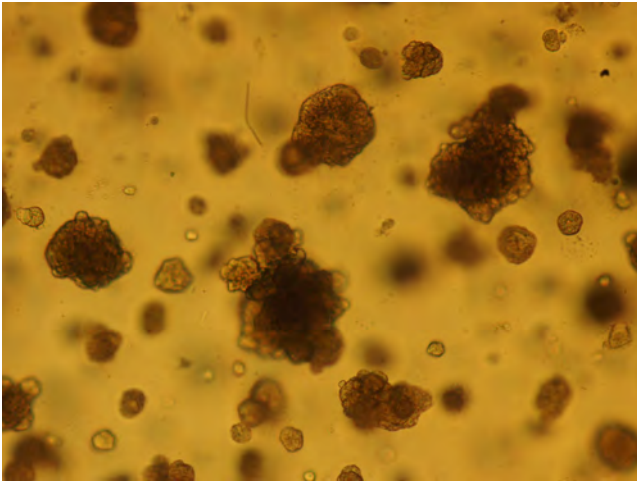

Irradiation JAG1

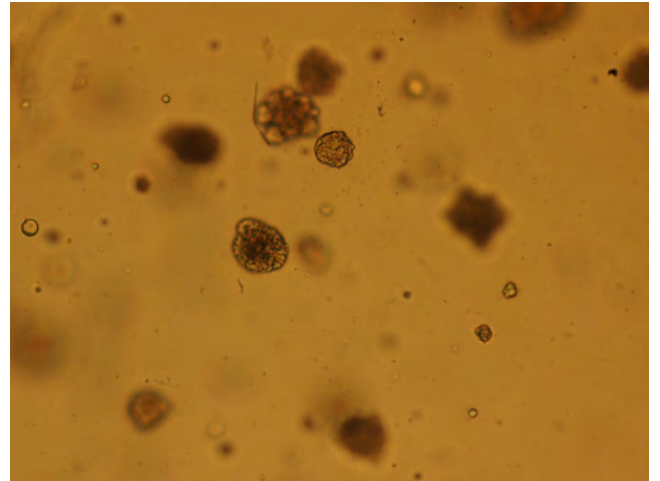

Irradiation DMSO

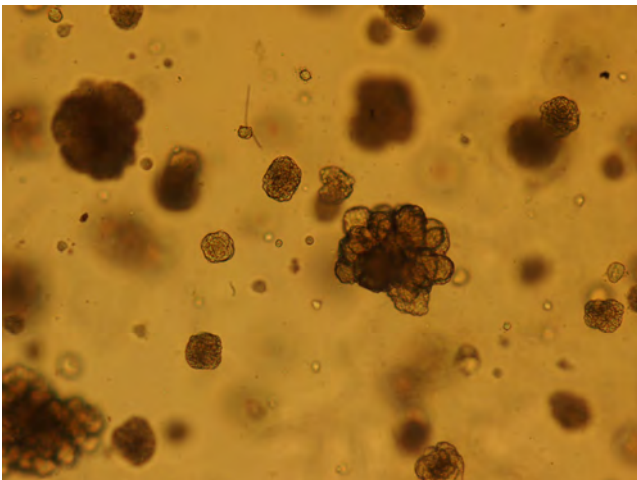

Irradiation JAG1

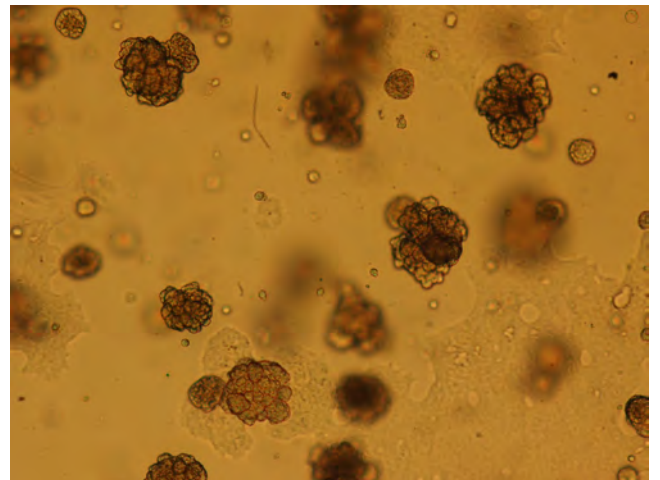

Supplement: Supplementary file 8 — Source data Fig. 4 [file 44318_2025_607_MOESM8_ESM.zip › Figure 4/4K/Microscopy irradiated mSGO_DMSO DBZ JAG1.pdf]

DMSO - 0h

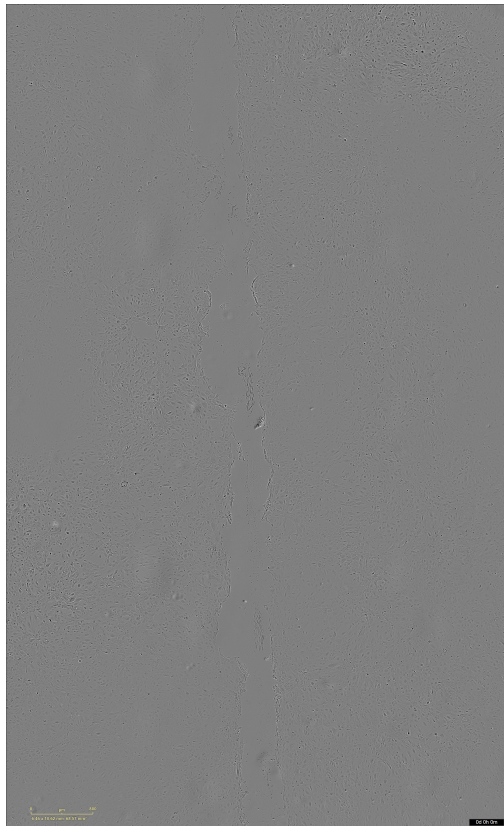

DMSO - 12h

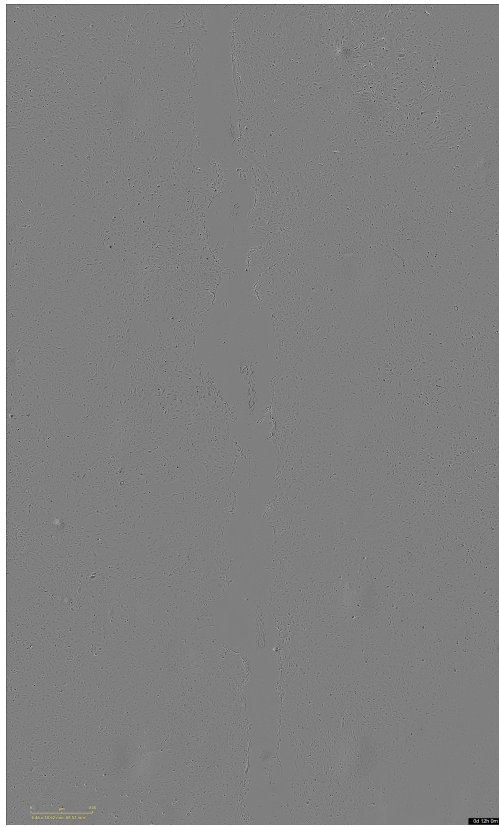

DMSO - 24h

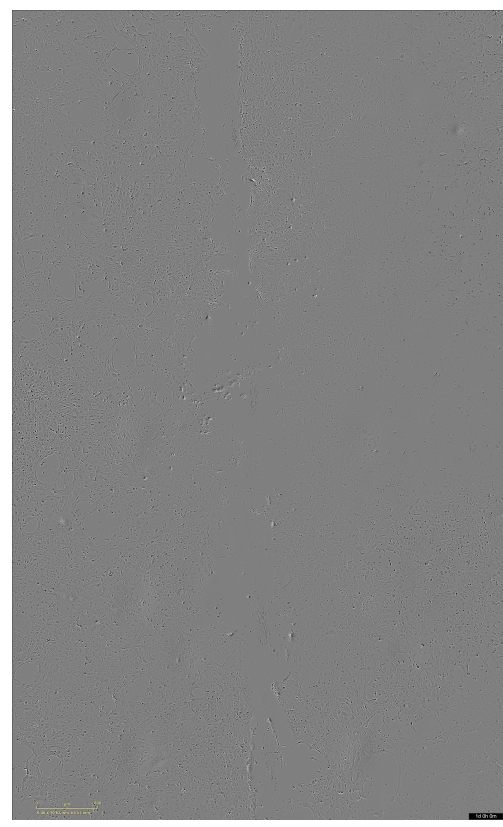

DBZ - 0h

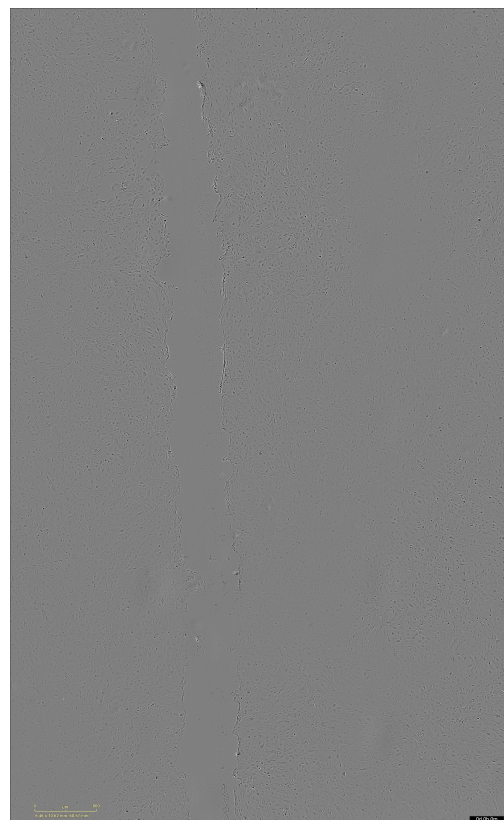

DBZ - 12h

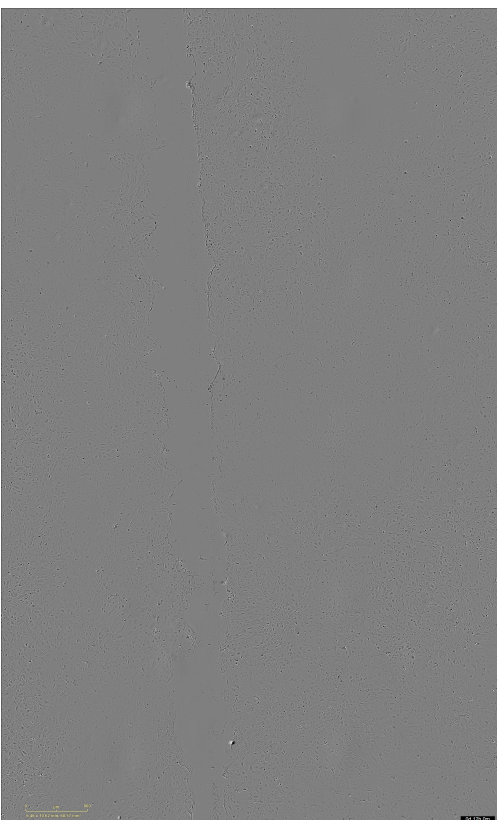

DBZ - 24h

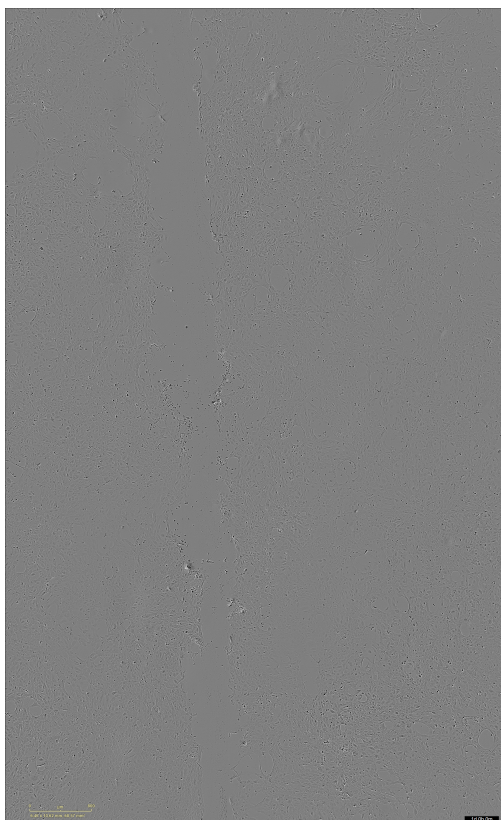

Supplement: Supplementary file 8 — Source data Fig. 4 [file 44318_2025_607_MOESM8_ESM.zip › Figure 4/4M/Microscopy scratch irradiated mSGO_DMSO DBZ.pdf]

DBZ

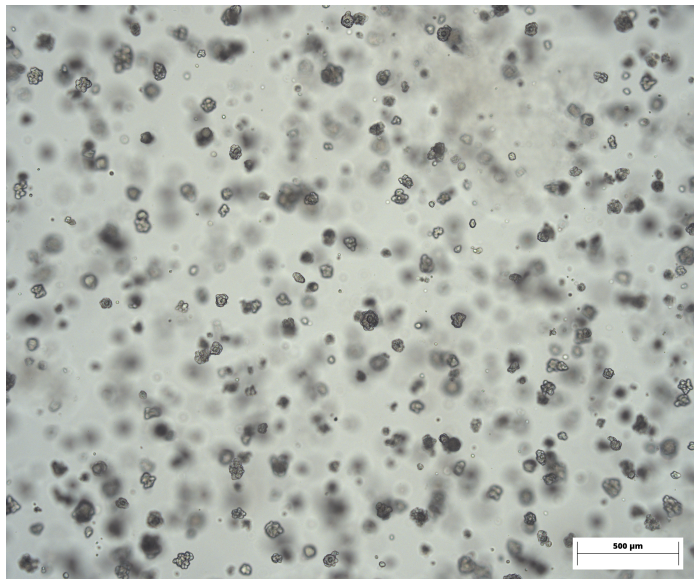

DMSO

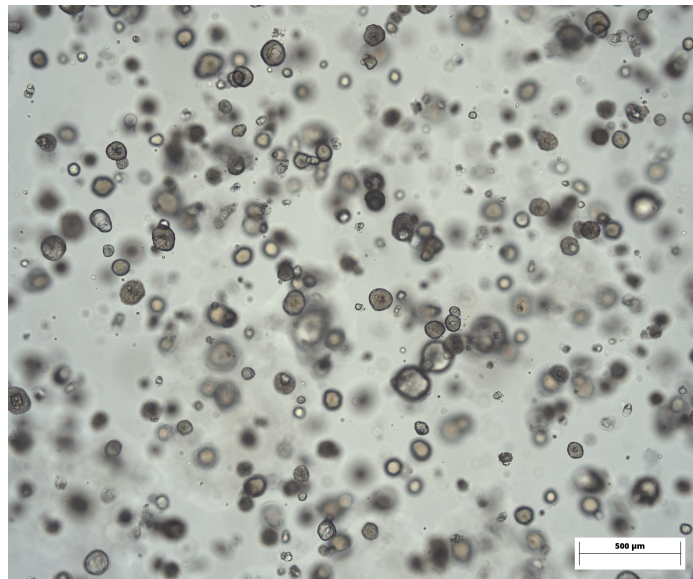

Supplement: Supplementary file 9 — Source data Fig. 5 [file 44318_2025_607_MOESM9_ESM.zip › Figure 5/5A/Microscopy mTGO_DMSO DBZ.pdf]

-DBZ day 0

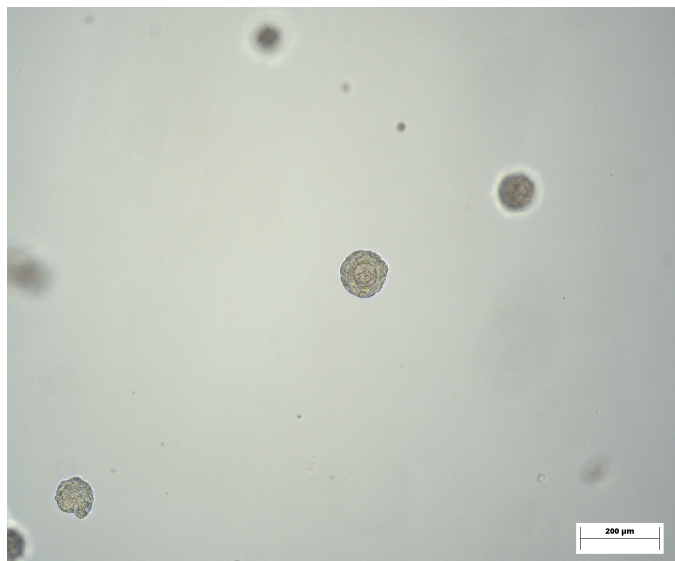

+DBZ day 0

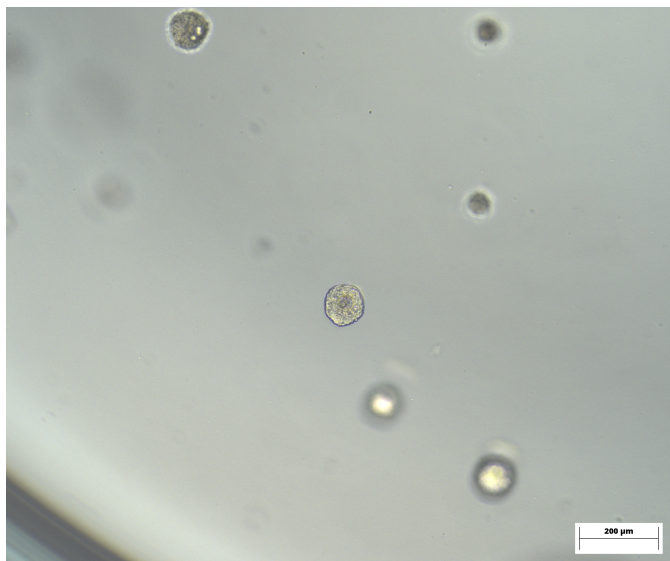

-DBZ day 4

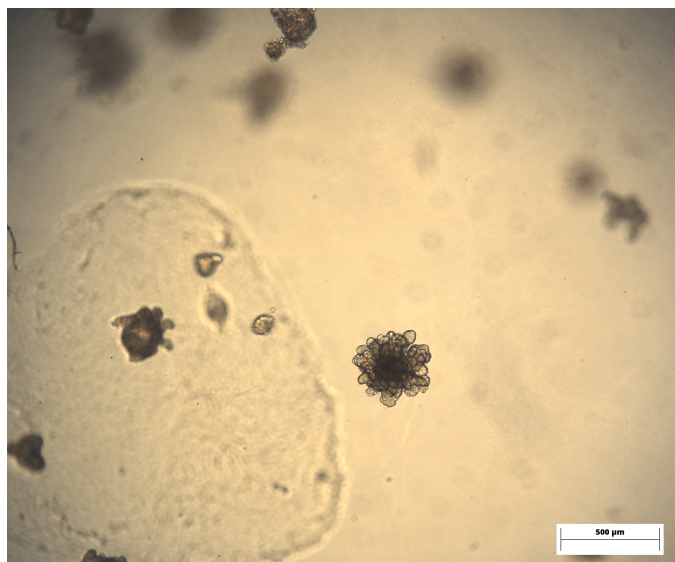

+DBZ day 4

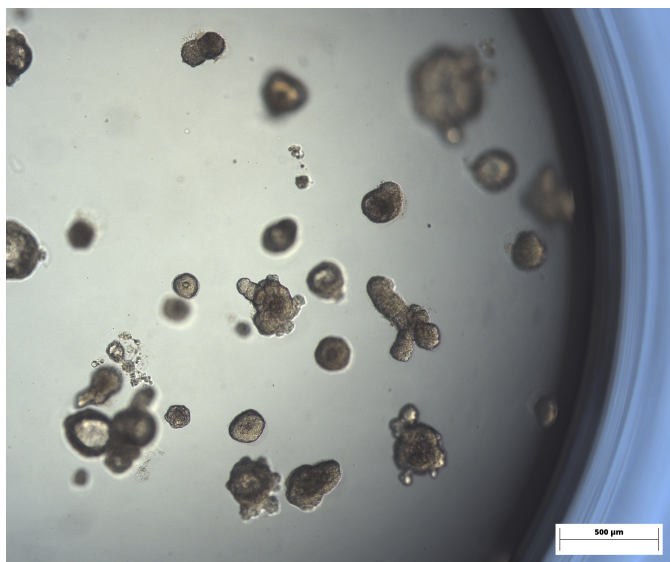

-DBZ day 8

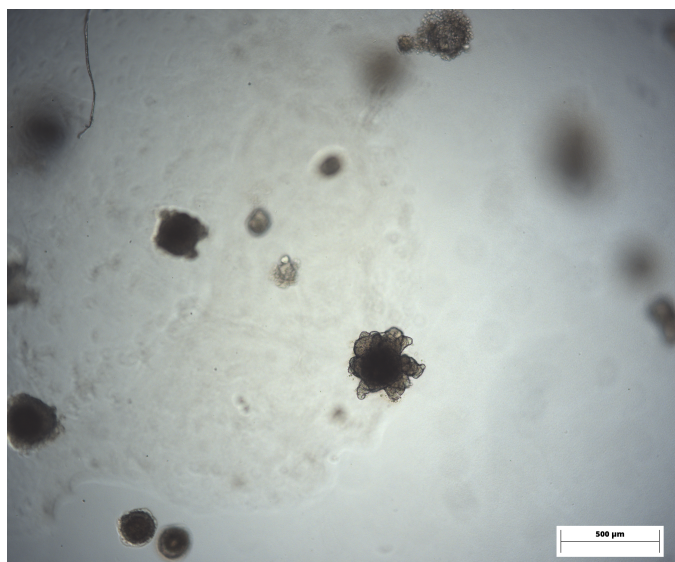

+DBZ day 8

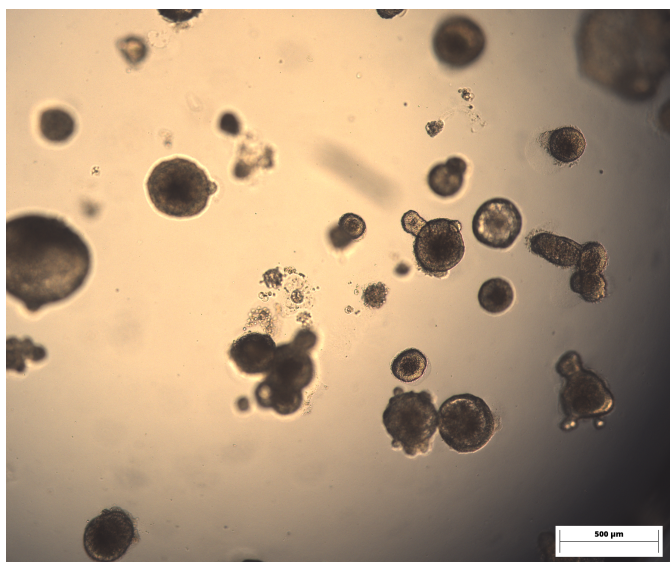

Supplement: Supplementary file 9 — Source data Fig. 5 [file 44318_2025_607_MOESM9_ESM.zip › Figure 5/5D/Microscopy differentiated mTGO.pdf]

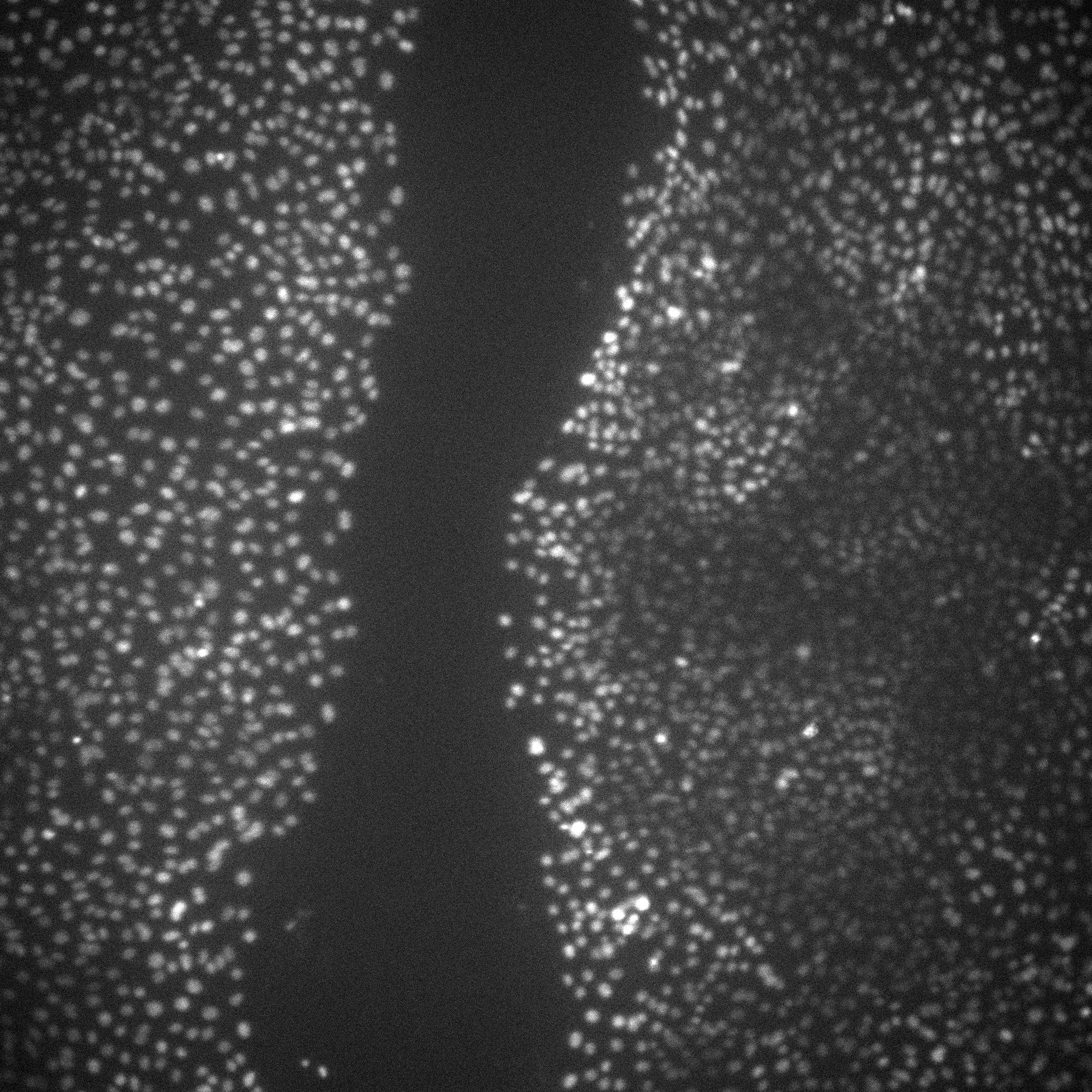

Supplement: Supplementary file 9 — Source data Fig. 5 [file 44318_2025_607_MOESM9_ESM.zip › Figure 5/5H/DMSO_DAPI.tif]

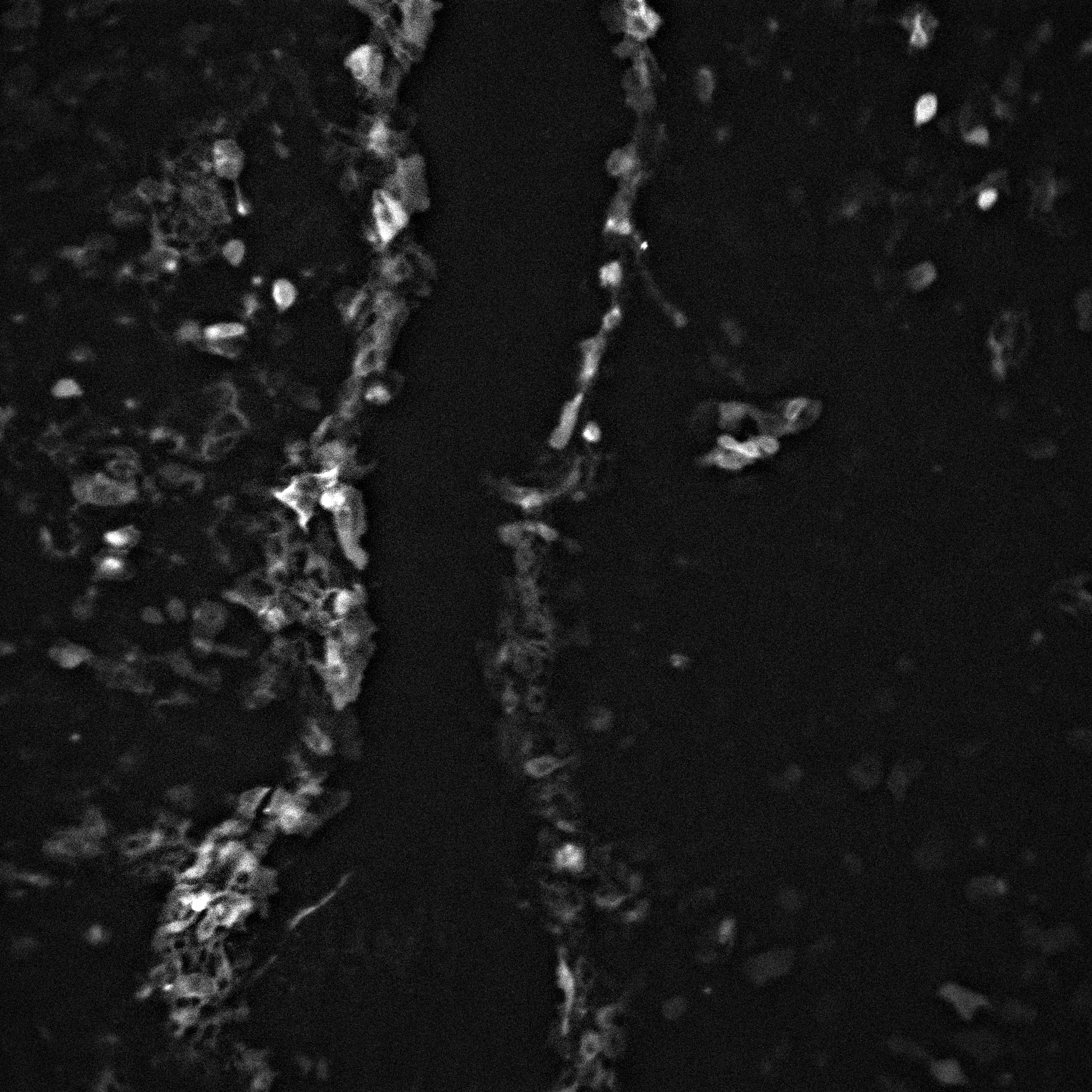

Supplement: Supplementary file 9 — Source data Fig. 5 [file 44318_2025_607_MOESM9_ESM.zip › Figure 5/5H/DMSO_CD44.tif]

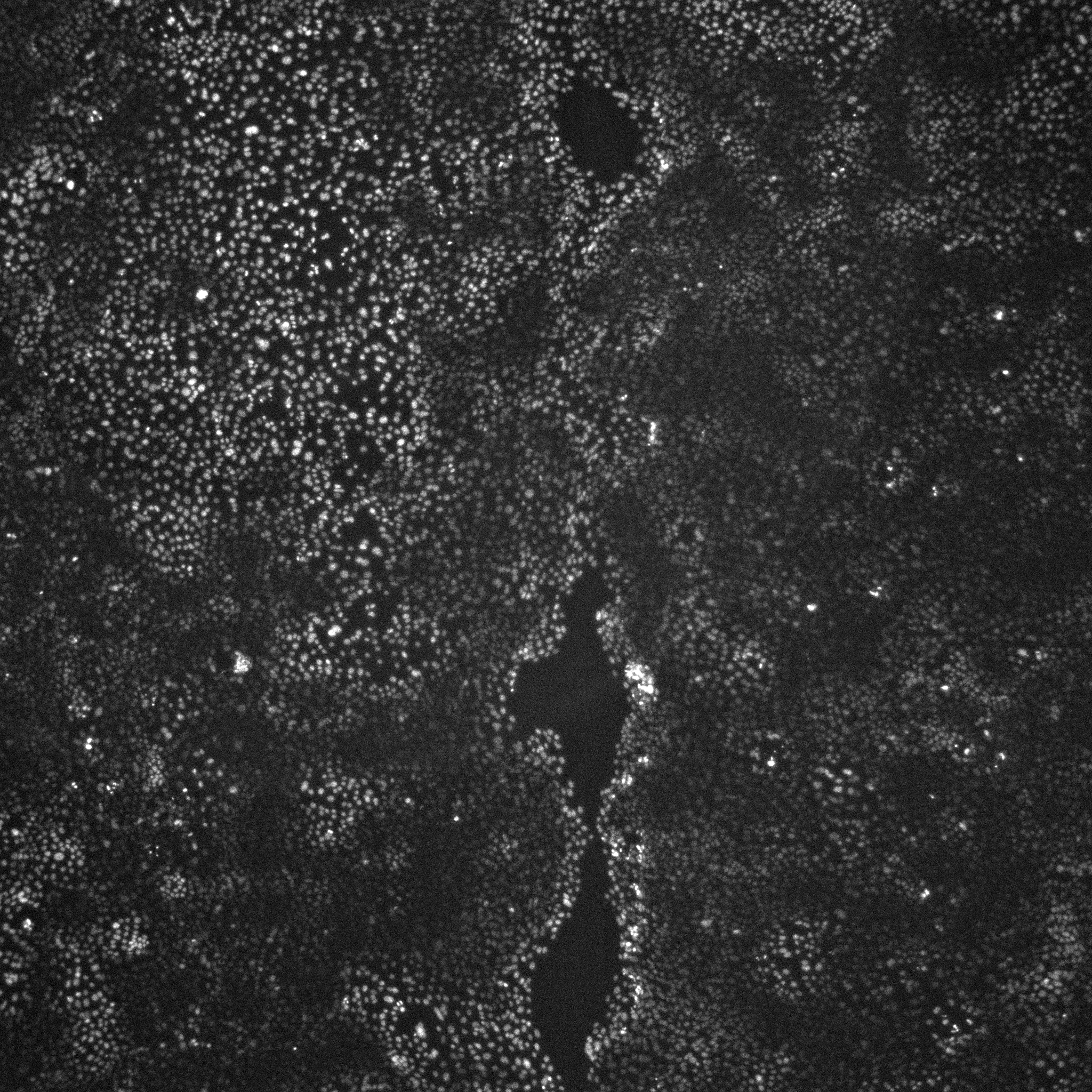

Supplement: Supplementary file 9 — Source data Fig. 5 [file 44318_2025_607_MOESM9_ESM.zip › Figure 5/5H/JAG1_DAPI.tif]

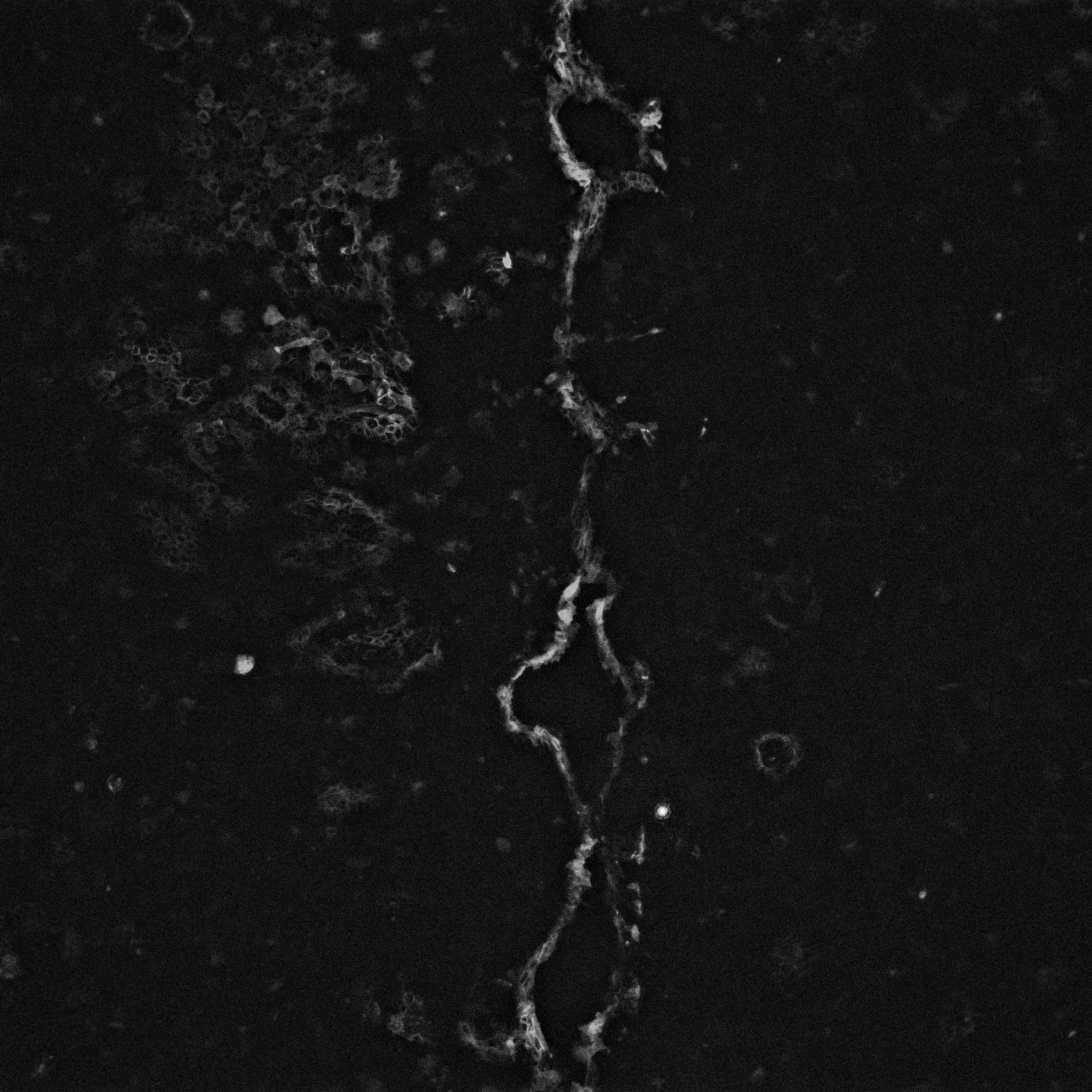

Supplement: Supplementary file 9 — Source data Fig. 5 [file 44318_2025_607_MOESM9_ESM.zip › Figure 5/5H/JAG1_DMSO.tif]

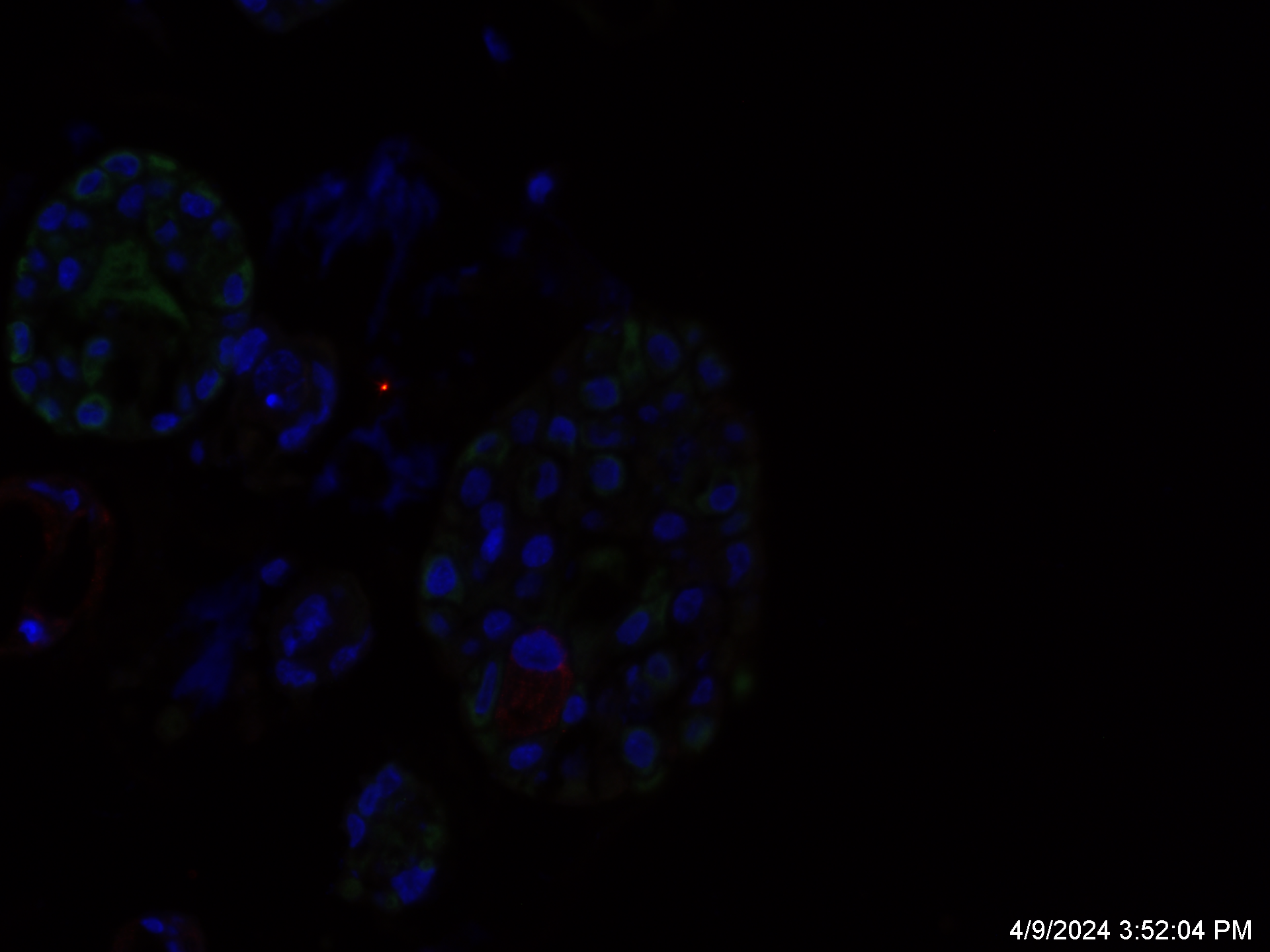

Supplement: Supplementary file 9 — Source data Fig. 5 [file 44318_2025_607_MOESM9_ESM.zip › Figure 5/5I/hSGO_DAPI_KRT14_KRT8_overlay.tif]

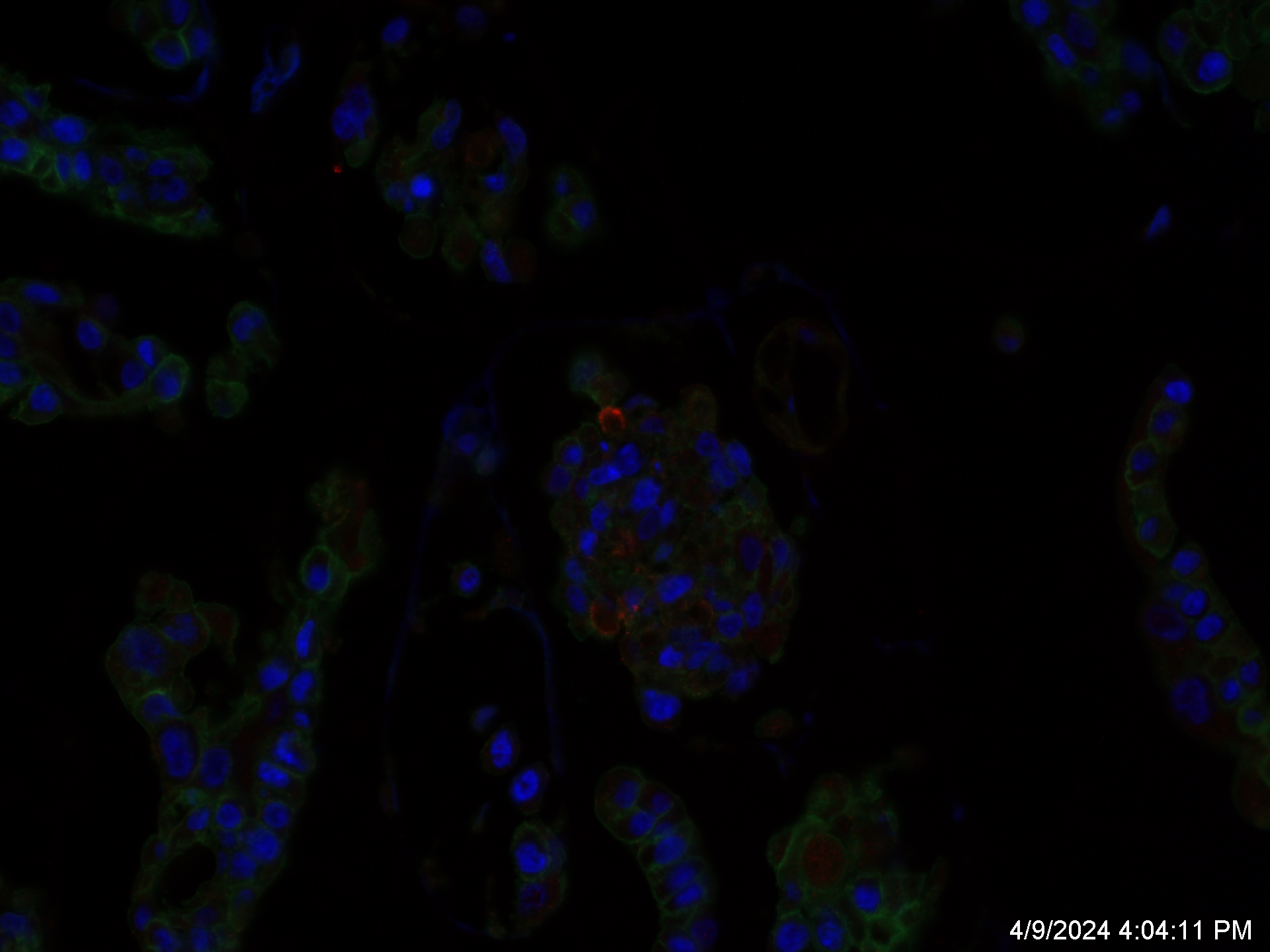

Supplement: Supplementary file 9 — Source data Fig. 5 [file 44318_2025_607_MOESM9_ESM.zip › Figure 5/5I/hSGO_DAPI_CD44_CD29_overlay.tif]

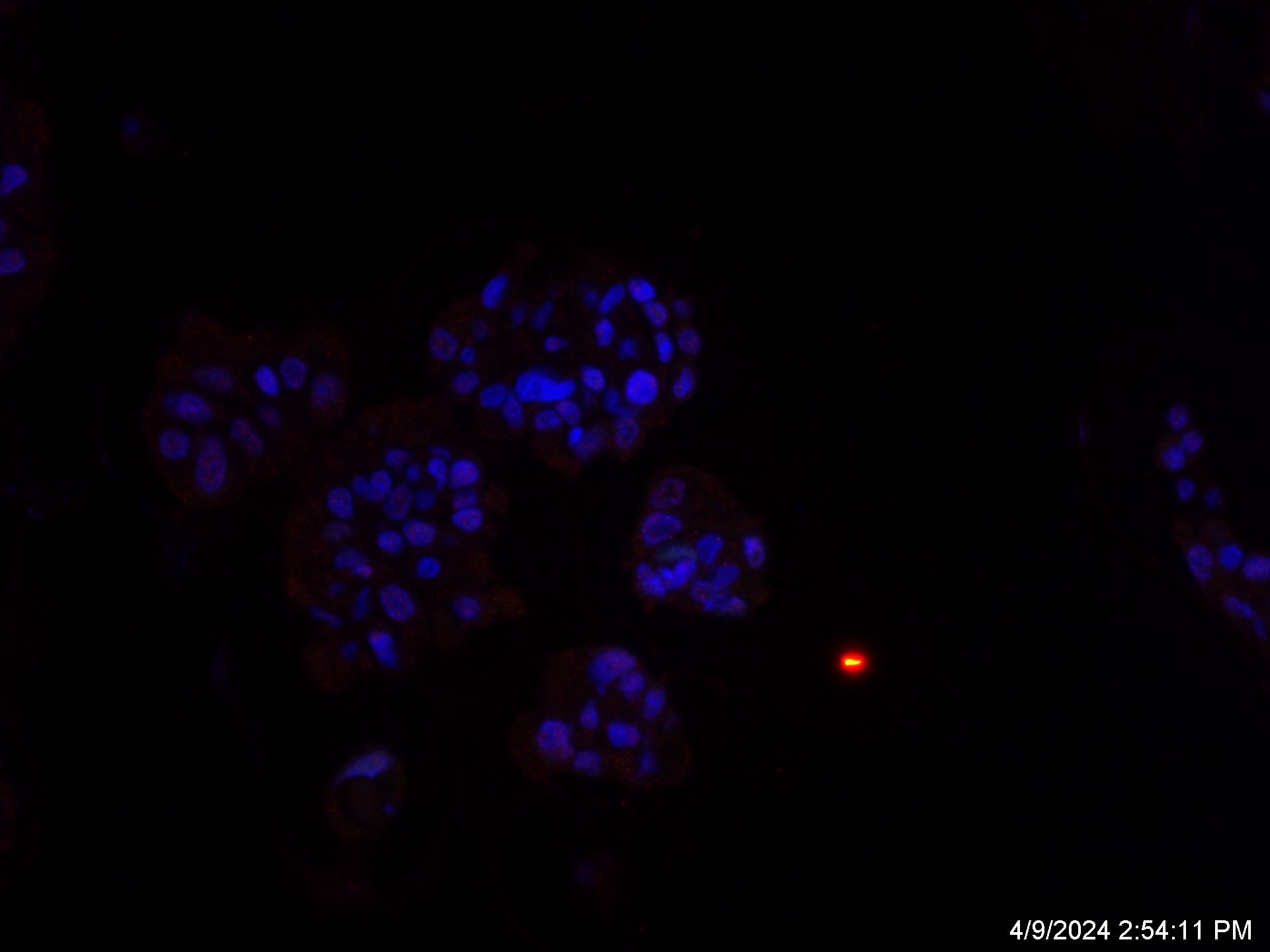

Supplement: Supplementary file 9 — Source data Fig. 5 [file 44318_2025_607_MOESM9_ESM.zip › Figure 5/5I/hSGO_DAPI_SOX9_CD164_overlay.tif]

DBZ

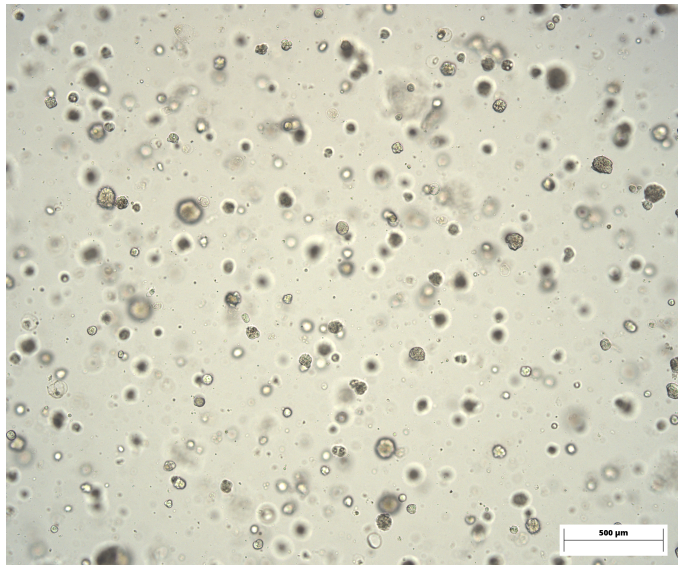

DMSO

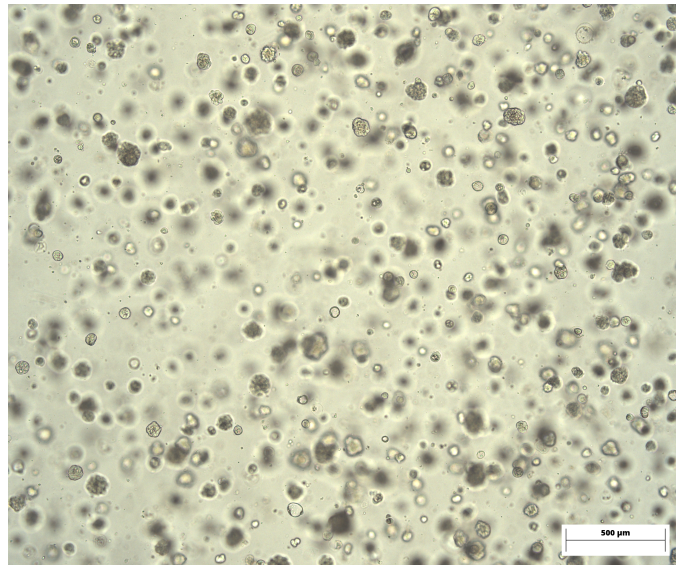

Supplement: Supplementary file 9 — Source data Fig. 5 [file 44318_2025_607_MOESM9_ESM.zip › Figure 5/5J/Microscopy hSGO_DMSO DBZ.pdf]

DBZ

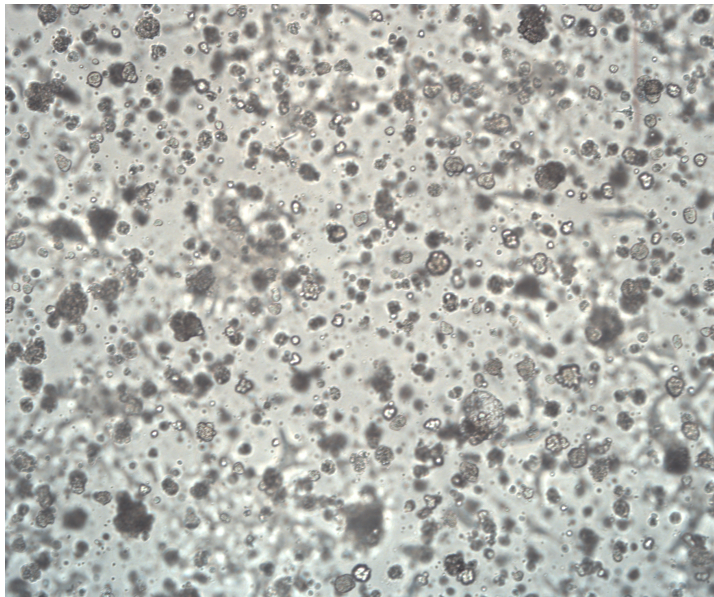

DMSO

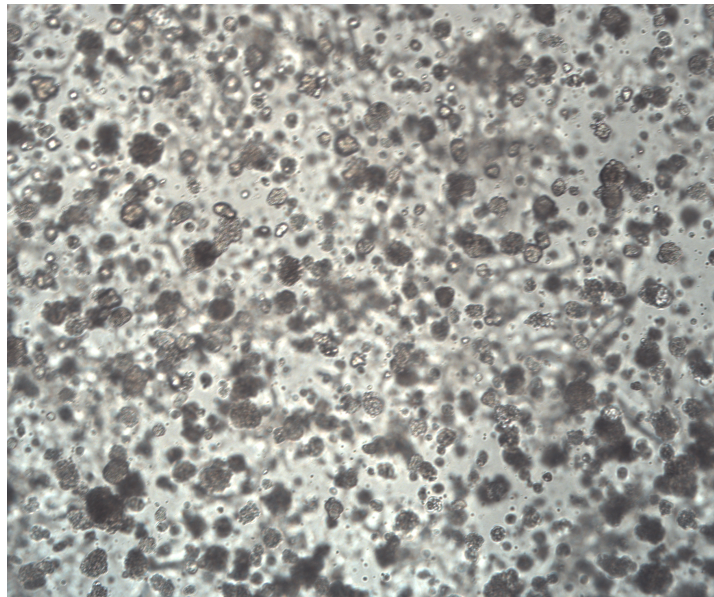

Supplement: Supplementary file 9 — Source data Fig. 5 [file 44318_2025_607_MOESM9_ESM.zip › Figure 5/5M/Microscopy hTGO_DMSO DBZ.pdf]

DMSO

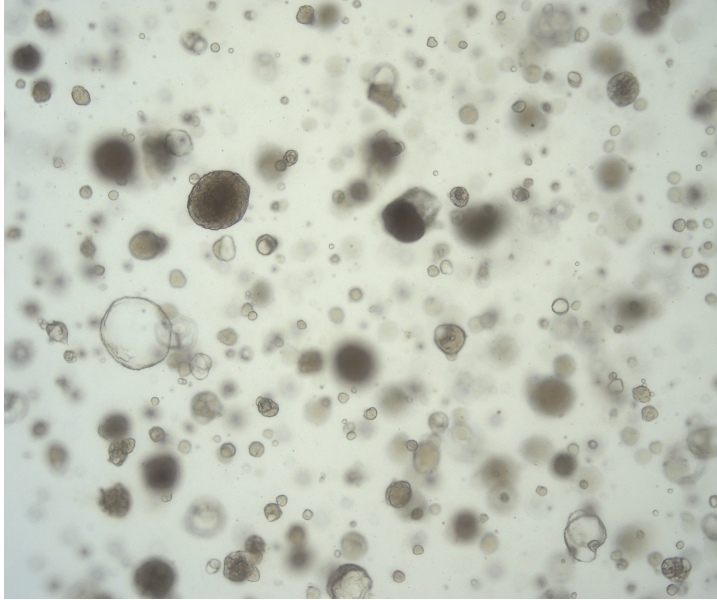

DBZ

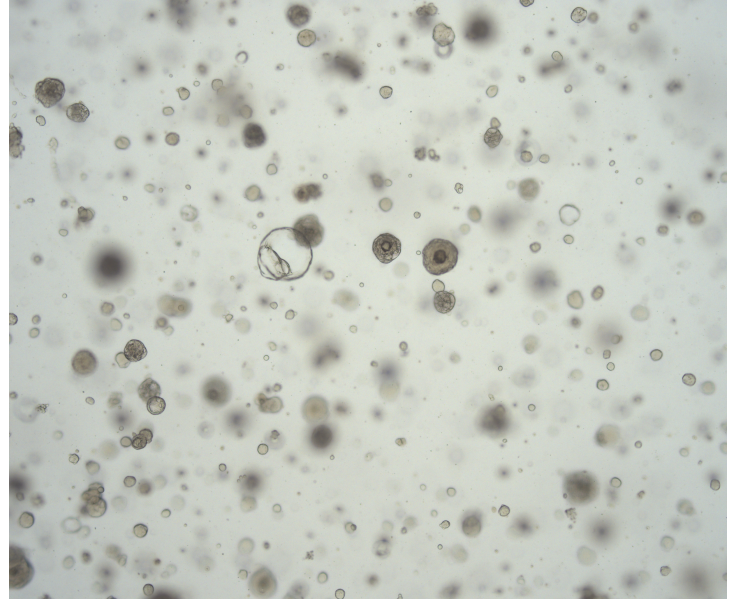

Supplement: Supplementary file 9 — Source data Fig. 5 [file 44318_2025_607_MOESM9_ESM.zip › Figure 5/5O/Microscopy hMGO_DMSO DBZ.pdf]
